# The current landscape of mRNA therapy and the strategies for mRNA purification and dsRNA removal

**DOI:** 10.1186/s12929-026-01277-4

**Published:** 2026-07-27

**Authors:** Jin-He Liu, Jian Zang, Jing-Ru Xu, Xue-Bin Ran, Min Su, Wang-Ming Zhang, Zhang-Wen Ge, Qiao-Yang Sun, Ling-Wen Ding

**Affiliations:** 1 Center for Tissue Engineering and Stem Cell Research, Translation Medicine Research Center，Guizhou Biomanufacturing Laboratory, Guizhou Medical University, Guiyang, 561113 China; 2 Department of Pathology, Yong Loo Lin School of Medicine, National University of Singapore, Singapore, 119074 Singapore; 3 Tianfu Yongxing Laboratory, Chengdu, 610213 China; 4 Cancer Science Institute of Singapore, National University of Singapore, Singapore, 119074 Singapore; 5 Central Laboratory, Beijing Jishuitan Hospital Guizhou Hospital, Guiyang, 550014 China; 6 Department of Laboratory Medicine, Guizhou Provincial People’s Hospital, Affiliated Hospital of Guizhou University, Guiyang, 550002 China; 7 Department of Neurology, National Neuroscience Institute, Singapore, Singapore

**Keywords:** mRNA therapy, dsRNA, mRNA purification, Innate immune response

## Abstract

Messenger RNA (mRNA) technology has emerged as a cornerstone in vaccine development and therapeutic applications, offering key benefits such as high potency, rapid scalability, and cost-effectiveness. The success of COVID-19 mRNA vaccines has underscored their efficacy and safety. However, residual byproducts generated during mRNA synthesis, such as unincorporated caps, nucleoside triphosphates (NTPs), DNA templates, enzymes, abortive transcripts, and double-stranded RNA (dsRNA), pose significant challenges to the clinical application of the RNA therapy. Among these, dsRNA is particularly problematic as it can activate various innate immune responses, suppress mRNA translation and potentially compromise the therapeutic efficacy of mRNA. Therefore, effectively removing dsRNA from in vitro synthesized mRNA is essential before its used in preclinical or clinical settings. In this review article, we provide a comprehensive overview of current mRNA development pipelines and ongoing clinical trials, and recent advances in mRNA purification techniques. Specifically, we focus on strategies for dsRNA removal, which can be broadly categorized into two approaches: (1) separating or removing dsRNA from in vitro transcription (IVT) mRNA products using methods such as RP-HPLC chromatography and cellulose-based purification; and (2) minimizing dsRNA formation during IVT by employing engineered RNA polymerase mutants, chaotropic agents, and magnetic beads, as well as modifying/optimizing DNA templates or RNA molecules to reduce dsRNA generation. We also discuss the advantages and limitations of these purification methods, the factors influencing the selection of purification strategies, and explore potential future directions for improving dsRNA purification technologies and their applications in mRNA-based therapeutics.

**Graphical abstract:**

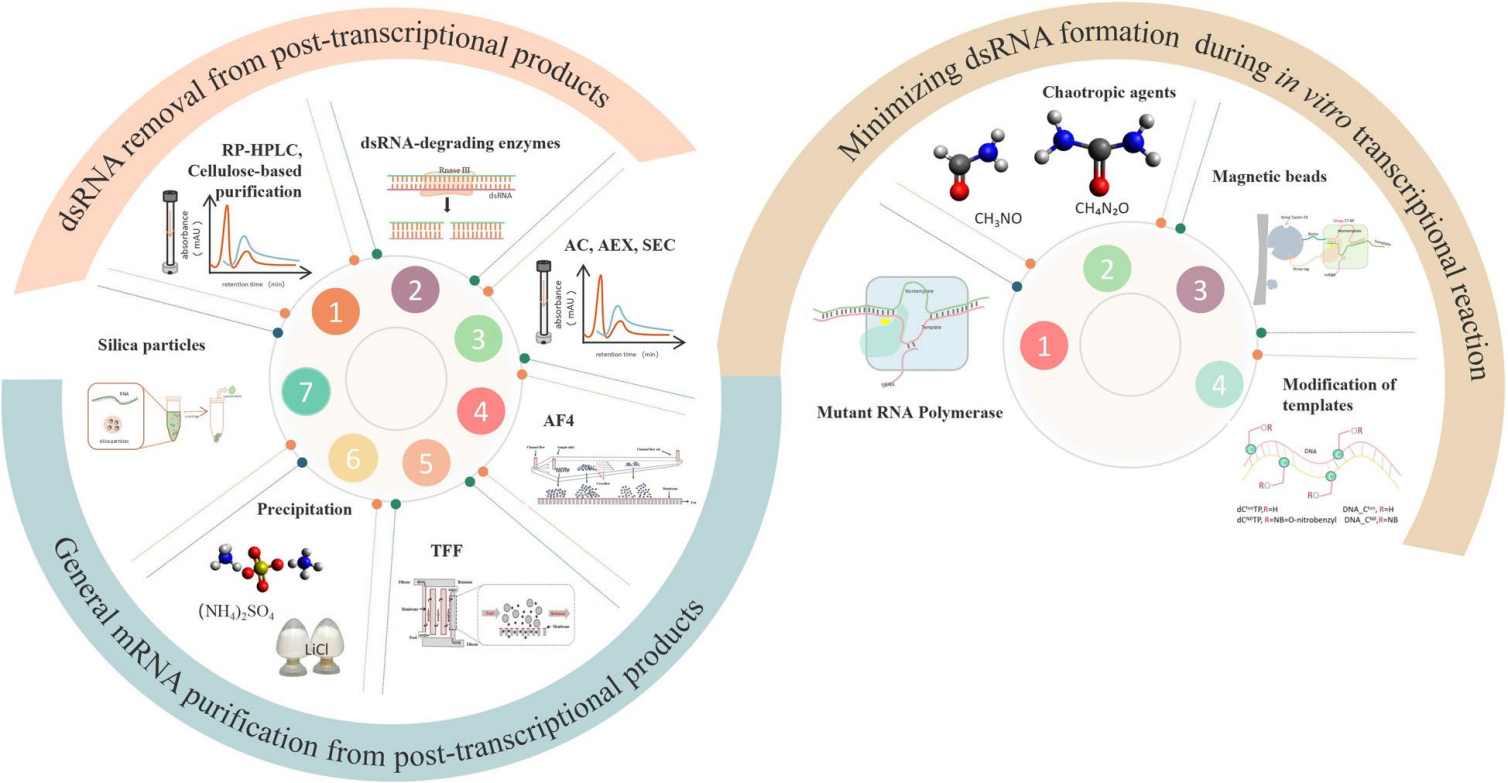

## Introduction

Over the past few decades, mRNA technology has evolved from a theoretical concept into a powerful tool for clinical applications. This technology has demonstrated remarkable potential in developing vaccines and therapeutics, including cancer treatments, gene therapy, and protein replacement therapies [1–5]. mRNA technology offers several advantages over traditional methods, including rapid development, flexible design, transient expression, and broad applicability [1]. Unlike DNA, which must first enter the nucleus for transcription, mRNA is directly translated into proteins in the cytoplasm. Notably, mRNA does not integrate into the host genome, thereby greatly reducing concerns about risks associated with gene integration and potential mutations [6].

**Fig. 1:**
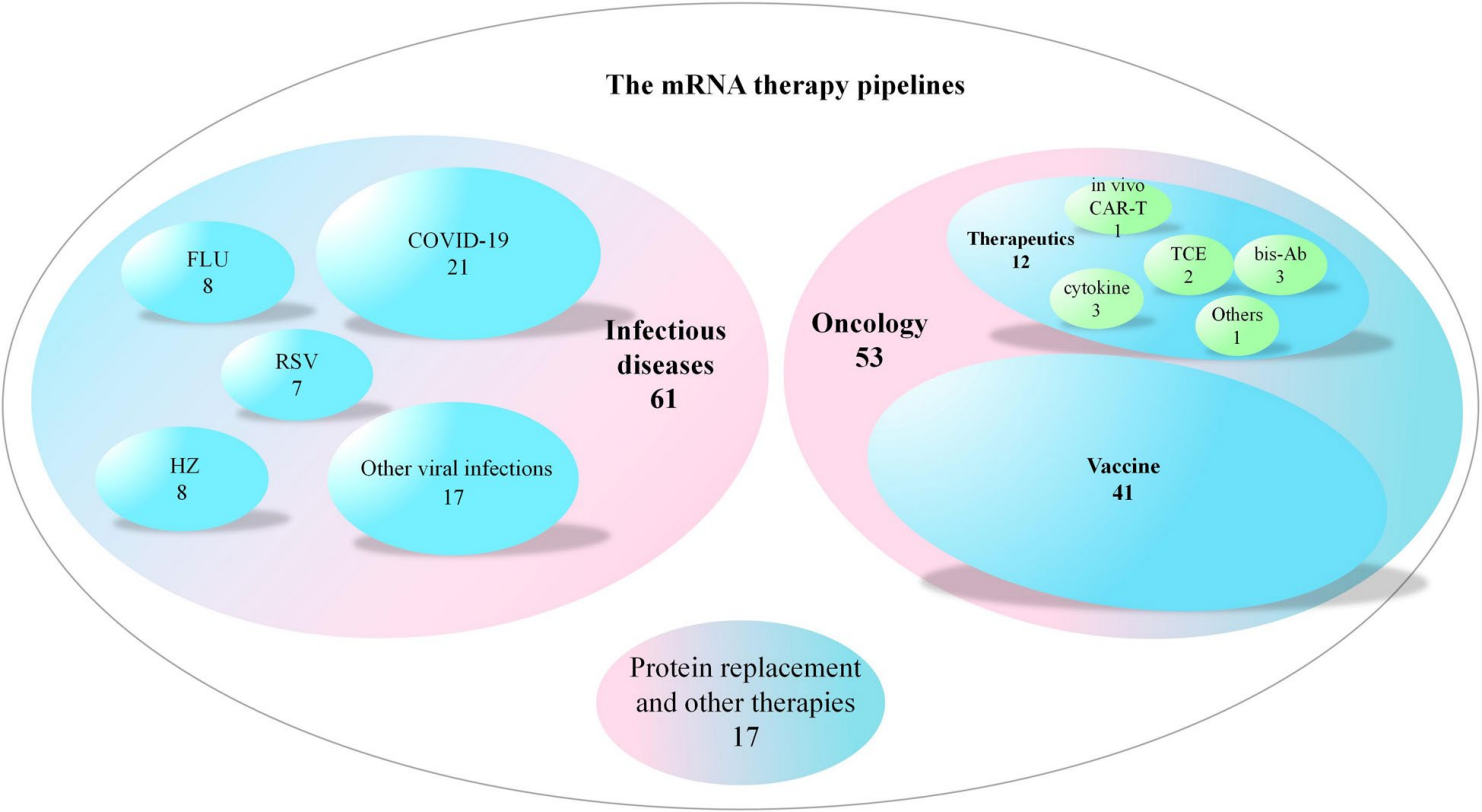
Overview of the development pipelines in mRNA therapies

The development and widespread use of COVID-19 mRNA vaccines represent the most significant achievement of mRNA technology, demonstrating strong effectiveness in reducing severe disease, hospitalization, and mortality during the pandemic [7, 8]. This milestone has catalyzed global efforts to develop mRNA vaccines and therapeutics for other diseases (Fig. 1), such as influenza (Flu) [9–11], respiratory syncytial virus (RSV) [12], herpes simplex virus (HSV) [13], monkeypox virus (MPXV) [14–16], and cancer treatment [17]. In Tables 1, 2 and 3, we summarize the current state of mRNA therapy in clinical applications, highlighting developments by various leading biotech companies and research institutions (Fig. 1). Many mRNA-based clinical applications are actively recruiting patients for Phase 1 or Phase 2 trials, and a significant number of mRNA therapies are expected to reach clinical use within the next decade.

**Table 1:** Current clinical development of mRNA-based therapies against infectious diseases

Application	Disease	Target	Company/Sponsor	Name	Clinical Trial	Status/Phase	Administration Routes
Vaccine	COVID-19	Spike protein	Moderna (USA)	Spikevax, mRNA-1273	NCT04470427, NCT06333704	Completed/Active, not recruiting/Phase3	I.M
Vaccine	COVID-19	Spike protein	BioNTech (Germany), G.Gennimatas General Hospital ( Greece)	COMIRNATY, BNT162b2	NCT07069309, NCT04756817, NCT04368728	Completed/Phase 3	I.M
Vaccine	COVID-19	Spike protein	Pfizer (USA)-BioNTech (Germany)	BNT162b2	NCT07300839, NCT07222384, NCT06923137	Active, not recruiting/Phase 3	I.M
Vaccine	COVID-19	Spike protein	Chulalongkorn University (Thailand) Technovalia (Australia)	ChulaCov19, BNA159	NCT04566276, NCT05605470	Completed/Phase 1/2	I.M
Vaccine	COVID-19	Segments of SARS-CoV-2 nucleocapsid, Membrane and ORF1ab proteins	BioNTech (Germany)	BNT162b4	NCT05541861	Completed/Phase 1	I.M
Vaccine	COVID-19	Spike protein	EyeGene (South Korea)	EG-COVID	NCT05188469	Completed/Phase 1/2a	I.M
Vaccine	COVID-19	Membrane-anchored RBD	University of Melbourne (Australia)	MIPSCo-mRNA-RBD-1	NCT05272605	Completed/Phase 1	I.M
Vaccine	COVID-19	Spike protein from the Omicron variant	GlaxoSmithKline (UK)	CV0501	NCT05477186	Completed/Phase 1	I.M
Vaccine	COVID-19	SARS-CoV-2 antigen	RVAC Medicines (USA)	RVM-V001	NCT05788185	Terminated/Phase 1b	I.M
Vaccine	COVID-19	Spike protein of Omicron variant BA.4/5	Abogen (China)	ABO1020	NCT05636319	Unknown status/Phase 2/3	I.M
Vaccine	COVID-19	Spike protein	Henry M. Jackson Foundation (USA)	IDCRP-154	NCT07287137	Active, not recruiting/Phase 4	I.M
Vaccine	COVID-19	N-terminal and receptor-binding domains of spike protein of XBB.1.5	Moderna (USA)	mNEXSPIKE^®^, mRNA-1283	NCT07089706	Recruiting/Phase 4	I.M
Vaccine	COVID-19	Spike protein	Moderna (USA)	DAN-COVID	NCT07279766	Active, not recruiting/Phase 4	I.M
Vaccine	COVID-19	Spike protein	Moderna (USA)	mRNA-1273-P403	NCT06585241	Recruiting/Phase 3b/4	I.M
Vaccine	COVID-19	Spike protein	PharmaJet (USA)	COVID-PJ-01	NCT06919796	Not yet recruiting/Phase 2	I.M. or I.D
Vaccine	COVID-19	Spike protein	CyanVac LLC (USA)	CVXGA	NCT06742281	Active, not recruiting/Phase 2b	I.N
Vaccine (sa-RNA)	COVID-19	Spike protein	HDT Bio (USA)	HDT-301	NCT05132907	Completed/Phase 1	I.M
Vaccine (sa-RNA)	COVID-19	Spike protein	Arcturus Therapeutics (USA), CSL (Australia)	ARCT-2303	NCT06279871	Completed/Phase 3	I.M
Vaccine (sa-RNA)	COVID-19	Spike protein, TCE regions Nuc, ORF3a/Membrane	Gritstone bio (USA)	GRT-R910	NCT05148962	Completed/Phase 1	I.M
Vaccine (sa-RNA)	COVID-19	Spike protein with the D614G mutation	Arcturus Therapeutics (USA), Vinbiocare (Vietnam), CSL (Australia), Fukushima Medical University School of Medicine (JP)	ARCT-154, KOSTAIVE^®^, zapomeran	NCT05012943, jRCTs021250024	Completed/Not recruiting/Phase 1/2/3a/3b	I.M
Vaccine (sa-RNA)	COVID-19	Full-length spike protein	Arcturus Therapeutics (USA), Fred Hutchinson Cancer Center (USA)	LUNAR-COV19,ARCT-021	NCT07390968	Not yet recruiting/Phase 2b	I.M
Vaccine (sa-RNA)	Influenza	HA and NA for four different influenza strains	Arcturus Therapeutics (USA)	ARCT-2138	NCT06125691	Completed/Phase 1	I.M
Vaccine	Influenza	HA of A/H1N1, A/H3N2, B/Victoria and B/Yamagata	Moderna (USA)	mRNA-1010	NCT04956575, NCT05827978	Completed/Phase 1/2/3	I.M
Vaccine	Influenza	HA and NA	Moderna (USA)	mRNA-1020, mRNA-1030	NCT05333289	Completed/Phase 1/2	I.M
Vaccine	Influenza	HA	Moderna (USA)	mRNA-1018	NCT05972174	Completed/Phase 1/2	I.M
Vaccine	Influenza	HA	Sanofi (France)	MRT5407	NCT05553301	Completed/Phase 1/2	I.M
Vaccine	Influenza	HA of 4 seasonally recommended strains	Pfizer (USA)	PF-07252220	NCT05540522	Completed/Phase 3	I.M
Vaccine	Influenza	Multivalent seasonal influenza vaccine	GlaxoSmithKline (UK)	GSK6498032A	NCT07204964	Active, not recruiting/Phase 2a	I.M
Vaccine	Influenza	Multivalent seasonal influenza vaccine	GlaxoSmithKline (UK)	GSK6479720A	NCT07121192	Completed/Phase 2a	I.M
Vaccine	RSV	pre-F protein	Sanofi (France)	SP-0256	NCT06251024	Completed/Phase 2b	I.M
Vaccine	RSV	pre-F protein of RSV-A2	Moderna (USA)	mRESVIA, mRNA-1345	NCT06143046, NCT06067230	Completed/Active, not recruiting/Phase 2/3	I.M
Vaccine	RSV	pre-F protein	AlphaNa (China)	AFN0205	CTR20252307	Recruiting/Phase 2	Not disclosed
Vaccine	RSV	pre-F protein	Abogen (China)	ABO1105	NCT07289542, NCT07289503	Active, not recruiting/Phase 1/2	Not disclosed
Vaccine	RSV	pre-F protein	CSPC (China)	SYS6016	CTR20243880, ChiCTR2400091478	Not yet recruiting/Phase 1	I.M
Vaccine	RSV	Not disclosed	Sinovac (China)	N/A	NCT07418229	Not yet recruiting/Phase 1	Not disclosed
Vaccine	RSV	Bivalent pre-F protein	InnoRNA (Shenxin Biotechnology, China)	IN006	NCT07128121, NCT06645665, CTR20252903, CTR20243326	Active, not recruiting/Phase 1/2	I.M
Vaccine	Herpes Zoster	Glycoprotein E	Abogen (China)	ABO1108	NCT07285278, NCT07285265	Active, not recruiting/Phase 1/2	Not disclosed
Vaccine	Herpes Zoster	Not disclosed	AlphaNa (China)	AFN1213	CTR20253368	Recruiting/Phase 1	Not disclosed
Vaccine	Herpes Zoster	Glycoprotein E	Moderna (USA)	mRNA-1468	NCT05701800	Active, not recruiting/Phase 1/2	I.M
Vaccine	Herpes Zoster	Glycoprotein E	Sinovac (China)	N/A	NCT07400003, CTR20254939	Recruiting/Phase 1/2	I.M
Vaccine	Herpes Zoster	Glycoprotein E	Rhegen (Regis Biotechnology, China)	RH110	CTR20254682, CTR20251808	Recruiting/Phase 1/2	I.M
Vaccine	Herpes Zoster	Glycoprotein E	InnoRNA (Shenxin Biotechnology, China)	IN001	NCT07205796	Not yet recruiting/Phase 2	I.M
Vaccine	Herpes Zoster	Glycoprotein E	CSPC (China)	SYS6017	NCT07354659, CTR20255261	Not yet recruiting/Recruiting/Phase 2	I.M
Vaccine (sa-RNA)	Herpes Zoster	Glycoprotein E	Immorna (China)	JCXH-105	NCT06581575	Active, not recruiting/Phase 2	I.M
Vaccine	Genital Herpes Simplex Virus Type 2	HSV-2 glycoproteins gB, gC and gD and ICP0, ICP4	Moderna (USA)	mRNA-1608	NCT06033261	Completed/Phase 1/2	I.M
Vaccine	Genital Herpes Simplex Virus Type 2	HSV-2 glycoproteins gC2, gD2, gE2	BioNTech (Germany)	BNT163	NCT05432583	Active, not recruiting/Phase 1	I.M
Vaccine	HIV	eOD-GT8 60mer, core-g28v2 60mer, N332-GT5 gp151	International AIDS Vaccine Initiative (USA)	IAVI G004, mRNA-1645	NCT06694753	Recruiting/Phase 1	I.M
Vaccine	HIV	HIV-1 Env gp150	NIAID (USA)	DV201P-RNA, DV202B1-RNA	NCT07390474	Recruiting/Phase 1	I.M
Vaccine	Zika Virus	prME from the Micronesia	Moderna (USA)	mRNA-1325	NCT03014089	Completed/Phase 1	I.M
Vaccine	Zika Virus	prME from the RIO-U1	Moderna (USA)	mRNA-1893	NCT04064905, NCT04917861	Completed/Phase 1/2	I.M
Vaccine	Mpox	Mpox antigens A35, B6, M1, H3	BioNTech (Germany)	BNT166a	NCT07379580	Recruiting/Phase 2	I.M
Vaccine	Mpox	Four conserved surface proteins of the monkeypox virus (MPXV) and other orthopoxviruses: M1, A29, A35, B6	Moderna (USA)	mRNA-1769	NCT05995275	Completed/Phase 1/2	I.M
Vaccine	Tuberculosis	Eight Mycobacterium tuberculosis antigens: Ag85A, Hrp1, ESAT-6, RpfD, RpfA, HbhA, M72, VapB47	BioNTech (Germany)	BNT164	NCT05547464	Active, not recruiting/Phase 1b/2a	I.M
Vaccine	Tuberculosis	Not disclosed	Rhegen (Regis Biotechnology, China)	RH119	ChiCTR2500109630	Recruiting/Phase 1	Not disclosed
Vaccine	Malaria	Plasmodium falciparum circumsporozoite protein	BioNTech (Germany)	BNT165	NCT05581641	Completed/Phase 1	I.M
Vaccine	CMV	Glycoprotein B, pentameric complex	Moderna (USA)	mRNA-1647	NCT05683457	Active, not recruiting/Phase 2	I.M
Vaccine	Chronic Hepatitis B Virus	HBV-positive tumor antigen HBx	West China Hospital (China)	WGc0201-HBV	NCT07051187	Recruiting/Phase 1	I.M
Vaccine	EBV	Four EBV envelope glycoproteins (gH, gL, gp42, gp220)	Moderna (USA)	mRNA-1189	NCT07478952, NCT05164094	Recruiting/Active, not recruiting/Phase 1/2	I.M
Vaccine	Lyme disease	OspA	Moderna (USA)	mRNA-1975, mRNA-1982	NCT05975099	Completed/Phase 1/2	I.M
Vaccine	Norovirus Acute Gastroenteritis	Trivalent vaccine, Capsid protein VP1 of GII.4, GI.3 and GII.3	Moderna (USA)	mRNA-1403	NCT06592794, NCT05992935	Active, not recruiting/Phase 1/2/3	I.M
Vaccine	Nipah Virus	Chimeric pre-F/G protein	Moderna and NIAID (USA)	mRNA-1215	NCT05398796	Completed/Phase 1	I.M

Disease and Target molecule: Cytomegalovirus (CMV), Epstein-Barr Virus (EBV), Hemagglutinin (HA), Human Immunodeficiency Virus (HIV), Premembrane and envelope E structural proteins (prME), Respiratory Syncytial Virus (RSV), Varicella-zoster Virus (VZV)

Administration Routes: Intramuscular (I.M), Intranasal (I.N), Intradermal (I.D)

Comirnaty, Spikevax, mRESVIA, KOSTAIVE and mNEXSPIKE have been approved by regulatory authorities. Regulatory and recruitment statuses were last verified on Jun. 19, 2026

**Table 2:** Current clinical development of mRNA-based therapies in oncology

Application	Disease	Target	Company/Sponsor	Name	Clinical Trial	Status/Phase	Administration Routes
Vaccine	Advanced/Metastatic Non-small cell lung cancer (NSCLC)	CLDN6, KK-LC-1, MAGE-A3, MAGE-A4, MAGE-C1, PRAME	BioNTech (Germany)	BNT116, LuCa-MERIT-1	NCT05142189	Recruiting/Phase 1	I.V
Vaccine	Advanced NSCLC with EGFR mutations	EGFR, EGFR L858R, EGFR T790M, EGFR-Ex19del	Abogen (China)	ABOR-2013	ChiCTR2500113656	Recruiting/Phase 1	I.M
Vaccine	Advanced Solid Tumors	Not disclosed	Moderna (USA)	mRNA-4359	NCT05533697	Recruiting/Phase 1/2	I.M
Vaccine	Recurrent/Refractory Multiple Myeloma (MM)	B-cell maturation antigen (BCMA)	Sichuan University (China)	WGb-0302	NCT07362732	Not yet recruiting/Early phase 1	Not disclosed
Vaccine	EBV-associated Tumors	EBV antigen	Xinqiao Hospital (China)	WGc-043	ChiCTR2500108428, NCT07349836	Not yet recruiting/Early Phase 1	Not disclosed
Vaccine	Cervical cancer	E6/E7 of HPV16 or HPV 18	CSPC (China)	SYS6026	CTR20253603, CTR20244614	Recruiting/Phase 1	I.D
Vaccine	UHNSCC, RHNC, MHNC	HPV16 E6/E7	BioNTech (Germany)	BNT113	NCT04534205	Recruiting/Phase 2/3	I.V
Vaccine	Cervical cancer, Cervical intraepithelial neoplasia (CIN)	HPV16/18	AlphaNa (China), Anke (China)	AFN0328	CTR20244013	Recruiting/Phase 1	I.M
Vaccine	Cervical cancer	HPV E6/E7	GeneLeap (China)	LY01620	CTR20243545	Recruiting/Phase 1	I.M
Vaccine	CIN	HPV 16/18	RinuaGene (China)	RG002	CTR20251020, NCT06273553	Not yet recruiting/Phase 1/2	I.M
Vaccine	CIN, Cervical cancer	E6/E7 of HPV 16	Newish (China)	NWRD09	NCT07047989, CTR20252163, NCT06741150	Recruiting/Phase 1	I.M
Vaccine	HBV-associated Hepatocellular Carcinoma (HCC)	HBV related antigens	West China Hospital (China)	N/A	NCT07077356	Recruiting/Phase 1	Not disclosed
Vaccine	HBV-associated HCC	HBV related antigens	West China Hospital (China)	WGc-0201	NCT07077369	Not yet recruiting/Phase 1	Not disclosed
Vaccine	Melanoma III or IV	NY-ESO-1, MAGE-A3, Tyrosinase, TPTE	BioNTech (Germany)	BNT111	NCT04526899, 2020-002195-12	Completed/Phase 2	I.V
Vaccine	Cutaneous Melanoma, Synovial Sarcoma	PRAME	Moderna (USA), Immatics (Germany)	mRNA-4203	NCT06946225	Recruiting/Phase 1	Not disclosed
Vaccine	KRAS-mutant Malignant Tumors	KRAS	Sichuan University (China)	N/A	NCT07004244	Recruiting/Phase 1	I.V
Vaccine	Advanced Solid tumors	Pan-Tumor Antigen (seven modified, full-length shared antigens)	Moderna (USA)	mRNA-4106	NCT06880549	Active, not recruiting/Phase 1	I.M
Vaccine	Advanced lung cancer and solid tumors with lung metastasis	Multiple shared, clinically validated TAAs	Cancer Hospital CAMS (China)	BMD006	NCT06928922	Recruiting/Early Phase 1	N.I
Vaccine	Squamous Non-Small Cell Lung cancer	Five shared TAAs	Everest Medicines (China)	EVM14	NCT07095868, NCT07614646	Recruiting/Not yet recruiting/Phase 1/2	I.M
Vaccine	Advanced Solid tumors	FAP	West China Hospital (China)	N/A	NCT07363369	Not yet recruiting/Phase 1	Not disclosed
Vaccine	Advanced Solid tumors	circFAM53B	Sun Yat-Sen Memorial Hospital (China)	circFAM53B-219aa mRNA	NCT07245901, ChiCTR2600117516	Not yet recruiting/Phase 1/2	I.M
Vaccine	Glioblastoma IDH Wildtype, Glioblastom WHO Grade 4	Not disclosed	Beijing Neurosurgical Institute (China)	GV-108/GV-907	NCT07520214	Enrolling by invitation/Early Phase 1	I.D
Vaccine	Advanced HCC	Nearly 20 HCC antigens	Peking Union Medical College Hospital (China)	ABOR2014, IPM511	NCT05981066	Phase 1	I.M
Vaccine	Glioblastoma	Eight TAA-derived epitopes	CureVac (Germany)	CVGBM, CV09050101	NCT05938387	Completed/Phase 1	I.M
Vaccine	Advanced or Recurrent Solid tumors	Personalized neoantigen	Everest Medicines (China)	EVM16	NCT06541639, EVM16CX01	Recruiting/Phase 1	Not disclosed
Vaccine	Colorectal cancer stage II\III, Resected pancreatic ductal adenocarcinoma (PDAC)	Personalized neoantigen	BioNTech (Germany), Genentech (USA)	BNT122, autogene cevumeran, RO7198457	NCT05968326, NCT04486378	Recruiting/Active, not recruiting/Phase 2	I.V
Vaccine	Advanced HCC	Personalized neoantigen	Zhejiang University (China)	iNeo-Vac-R01	NCT06995105	Recruiting/Phase 1/2	S.C
Vaccine	KRAS-mutant malignancies	Personalized neoantigen	West China Hospital of Sichuan University (China)	N/A	ChiCTR2500103833	Not yet recruiting	Not disclosed
Vaccine	KRAS -Mutated solid tumors	Five KRAS mutant antigens	Ruijin Hospital (China)	ABO2102	NCT06577532	Recruiting/Early Phase 1	I.M
Vaccine	Pancreatic cancer	Personalized neoantigen	Jinling Hospital (China)	SJ-Neo006	NCT06326736	Recruiting/Phase 1	Not disclosed
Vaccine	Advanced pancreatic cancer	Personalized neoantigen	Ruijin Hospital (China)	mRNA-0217/S001	NCT05916261	Recruiting/Early Phase 1	Not disclosed
Vaccine	Advanced solid tumors	Personalized neoantigen	Regenelead (China), Fudan University (China)	RGL-270	NCT07348042, CTR20251935	Recruiting/Phase 1	Not disclosed
Vaccine	Advanced or Metastatic Solid Tumors	Personalized neoantigen	Cancer Hospital Chinese Academy of Medical Science (China)	RH125	NCT07182435	Not yet recruiting/Early Phase 1	Not diclosed
Vaccine	Advanced Solid tumors	Personalized neoantigen	Rongcan Biotech (China), Shanghai Sixth People's Hospital (China)	ZY008	ChiCTR2600117499	Not yet recruiting/N/A	Not disclosed
Vaccine	Advanced/Recurrent or stage II/III Solid tumor	Personalized neoantigen	AlphaNa (China)	AFN18	ChiCTR2400090447	Recruiting/Early Phase 1	Not disclosed
Vaccine	NSCLC, Renal cell carcinoma, Bladder cancer, Melanoma, Non-Muscle Invasive Bladder Neoplasms	Up to 34 personalized neoantigens	Moderna (USA), Merck & Dohme LLC (USA)	mRNA-4157, V940	NCT06077760, NCT07513376, NCT07221474, NCT06623422, NCT06307431, NCT06305767, NCT05933577, NCT03313778, jRCT2061240063, NCT03897881, NCT06961006, NCT06833073	Recruiting/Active, not recruiting/Phase 1/2/3	I.M
Vaccine	Advanced intrahepatic Cholangiocarcinoma, Advanced/resectable pancreatic cancer, Biliary malignant tumors, Advanced or resected digestive system neoplasms	Personalized neoantigen	Zhejiang University (China), Sir Run Run Shaw Hospital (China)	iNeo-Vac-R01	NCT06956716, NCT07368803, NCT06888648, NCT06888674, NCT06026774, NCT06019702	Recruiting/Not yet recruiting/Phase 1/2	S.C
Vaccine	R/R B-cell Non-Hodgkin's Lymphoma (B-NHL)	Personalized neoantigen	Ruijin Hospital (China)	XP-006	NCT07334574	Not yet recruiting/Phase 1	Not disclosed
Vaccine	Gastric Cancer (GC), Biliary Cancer, PDAC, HCC	Personalized neoantigen	Beijing GoBroad Hospital (China), Nanjing Drum Tower Hospital (China), NeoCura (China)	XH-001	NCT07329894, NCT07298200, NCT07594964	Recruiting/Phase 1	Not disclosed
Vaccine	Gastrointestinal tumors	Personalized neoantigen	Ruijin Hospital (China)	PCV-GSTT	NCT07067385	Recruiting/Phase 1	Not disclosed
Vaccine	Acute Myeloid Leukemia	Personalized neoantigen	Shanghai Jiao Tong University School of Medicine (China)	XP-005	NCT06980155	Recruiting/Early Phase 1	Not disclosed
Therapeutics (in vivo CAR-T)	Hematological Malignancies	CD20	The 923rd Hospital of Joint Logistics Support Force of People's Liberation Army (China)	N/A	NCT07362602	Not yet recruiting/Early Phase 1	Not disclosed
Therapeutics (in vivo CAR-T)	Mesothelin-positive Advanced Malignant Solid Tumors	Mesothelin	UTC Therapeutics (USA)	UCMYM802	NCT06256055	Unknown status/Phase 1	I.V
Therapeutics (bispecific antibody)	EpCAM positive advanced malignant solid tumors	EpCAM/CD3	IntraAb (USA)	PMC2129G12	NCT07587879, ChiCTR2500103982	Not yet recruiting/Phase 1/2a	I.T
Therapeutics (bispecific antibody)	CLDN6-positive Solid Tumors	CLDN6, CD3	BioNTech (Germany)	BNT142	NCT05262530, 2021-005481-18	Terminated/Phase 1/2a	I.V
Therapeutics (bispecific antibody)	Advanced Gastric cancer	EpCAM/CD3	Abogen (China), Ruijin Hospital (China)	ABO2202	ChiCTR2400088554	Not yet recruiting/N/A	I.P
Therapeutics (T-cell engagers)	Relapsed or Refractory Multiple Myeloma (RRMM)	BCMA, FcRH5, GPR5d	Moderna (USA)	mRNA-2808	NCT07116616	Recruiting/Phase 1/2	I.V
Therapeutics (T-cell engagers)	R/R B-NHL, Autoimmune diseases	CD19/CD3	Abogen (China), Ruijin Hospital (China)	ABO2203	NCT07072169, NCT06747156	Recruiting/Phase 1/Early Phase 1	S.C. or I.V
Therapeutics (cytokine therapies)	Advanced Malignancies	IL-23, IL-36γ, OX40L	Moderna (USA)	mRNA-2752	NCT03739931	Completed/Phase 1	I.T
Therapeutics (cytokine therapies)	Advanced Solid tumors	IL-12	Abogen (China)	ABO-2011	NCT06088004	Recruiting/Phase 1/2	I.T
Therapeutics (cytokine therapies, sa-RNA)	Advanced Solid tumors	IL-12	Immorna (China)	JCXH-211	NCT05727839, CTR20230136	Completed/Phase 1	I.T
Therapeutics	Advanced solid tumors	CLDN6 (used together with CLDN6 Car-T therapy)	BioNTech Cell & Gene Therapies GmbH (Germany)	BNT211-01	NCT04503278	Active, not recruiting/Phase 1	I.V
Therapeutics (in vivo CAR-T)	Relapsed and Refractory immune related diseases, B-cell malignant tumors	CD19	West China Hospital (China), Sichuan University (China)	WGb-0301	NCT07349823, NCT07332663	Not yet recruiting/Early Phase 1	Not disclosed

Disease and Target molecule: B-cell maturation antigen (BCMA), B-cell Non-Hodgkin's Lymphoma (B-NHL), Cervical Intraepithelial Neoplasia (CIN), Hepatocellular Carcinoma (HCC), Isocitrate Dehydrogenase (IDH), Metastatic Head and Neck Cancer (MHNC), Non-small cell lung cancer (NSCLC), Pancreatic Ductal Adenocarcinoma (PDAC), Recurrent Head and Neck Cancer (RHNC), Unresectable Head and Neck Squamous Cell Carcinoma (UHNSCC)

Administration Routes: Intratumoral (I.T), Intravenous (I.V), Intramuscular (I.M), Intradermal (I.D), Intraperitoneal (I.P), Inhalation/Nebulizing inhaled (N.I), Subcutaneous (S.C)

Regulatory and recruitment statuses were last verified on June 19, 2026

**Table 3:** Current clinical development of mRNA therapies in protein replacement therapy and other diseases

Application	Disease	Target	Company/Sponsor	Name	Clinical Trial	Status/Phase	Administration Routes
Therapeutics	OTC deficiency	Ornithine Transcarbamylase (OTC)	Arcturus Therapeutics (USA)	ARCT-810	NCT06488313	Recruiting/Phase 2a	I.V
Therapeutics	Cystic fibrosis, CFTR gene mutation	CFTR	Arcturus Therapeutics (USA)	ARCT-032, LUNAR-CF	NCT06747858	Recruiting/Phase 2	N.I
Therapeutics	Cystic fibrosis	CFTR	Vertex (USA)	VX-522	NCT05668741	Active, not recruiting/Phase 1/2	O.I
Therapeutics	Chronic heart failure	Rel2- vlk	Moderna (USA)	mRNA-0184	NCT05659264	Completed/Phase 1	I.V
Therapeutics	Propionic acidemia (PA)	Propionyl-CoA carboxylase α/β	Moderna (USA)	mRNA-3927	NCT04159103	Recruiting/Phase 1/2	I.V
Therapeutics	Methylmalonic acidemia (MMA)	Methylmalonyl- CoA mutase	Moderna (USA)	mRNA-3705	NCT05295433	Recruiting/Phase 1/2	I.V
Therapeutics	Glycogen storage disease type 1a	Glucose-6-phosphatase	Moderna (USA)	mRNA-3745	NCT05095727	Active, not recruiting/Phase 1/2	I.V
Therapeutics	Lower limb ischemic disease	human hepatocyte growth factor	Regenelead (China)	RGL-2102	ChiCTR2600118847	Active, not recruiting/Phase 2	I.M
Therapeutics	Diabetic foot ulcer	human hepatocyte growth factor	Regenelead (China)	RGL-2102	ChiCTR2500113376	Not yet recruiting/Phase 2/3	T.C
Therapeutics	Refractory gout	Urate oxidase	Innorna (China)	IN026	NCT07587684	Not yet recruiting/Phase 1	I.V
Therapeutics	ATTR-CM/ATTR-PN	Transthyretin	Zhejiang University (China), YolTech (China)	YOLT-201	NCT06082050, NCT06539208	Recruiting/Early Phase 1/Phase 1/2a	I.V
Therapeutics	Wilson disease	ATP7B	DSciLab (China)	DSL101	NCT07240896	Recruiting/Early Phase 1	I.V
Therapeutics	Hypoparathyroidism	PTH	Peking Union Medical College Hospital (China)	XH-02	NCT07530705, NCT07540286	Recruiting/Phase 1/2	S.C
Therapeutics	Duchenne muscular dystrophy	Full-length dystrophin	Siponuoyin (China)	SPOT-03	NCT07188012	Recruiting/Early Phase 1	I.V
Therapeutics	Asthma	Interferon lambda ligand	Ethris GmbH (Germany)	ETH47	NCT07059767, ISRCTN21576805	Recruiting/Phase 2a	I.N
Therapeutics	Acne	Pathogenic antigens on specific strains of Cutibacterium acnes	Sanofi (France)	SP0268	NCT07013747, NCT06316297	Recruiting/Phase 1/2	I.M
Therapeutics (CAR-T)	Myasthenia gravis, Systemic lupus erythematosus	BCMA	CSPC (China)	SYS6020	CTR20244175, CTR20244320, NCT06688435	Recruiting/Phase 1	I.V

Disease and Target molecule: Transthyretin Amyloidosis Polyneuropathy (ATTR-PN), Transthyretin Amyloidosis Cardiomyopathy (ATTR-CM), Relapsed/Refractory B-cell maturation antigen (BCMA), Cystic fibrosis transmembrane conductance regulator (CFTR), Glycogen Storage Disease Type 1a (GSD1a), Methylmalonyl-coenzyme A (CoA) mutase (MUT), Ornithine transcarbamylase (OTC)

Administration Routes/Methods: Intravenous (I.V), Intramuscular (I.M), Intranasal (I.N), Nebulizing inhaled (N.I), Oral inhalation (O.I), Subcutaneous (S.C), Topical (T.C)

Regulatory and recruitment statuses were last verified on June 18, 2026

**Fig. 2:**
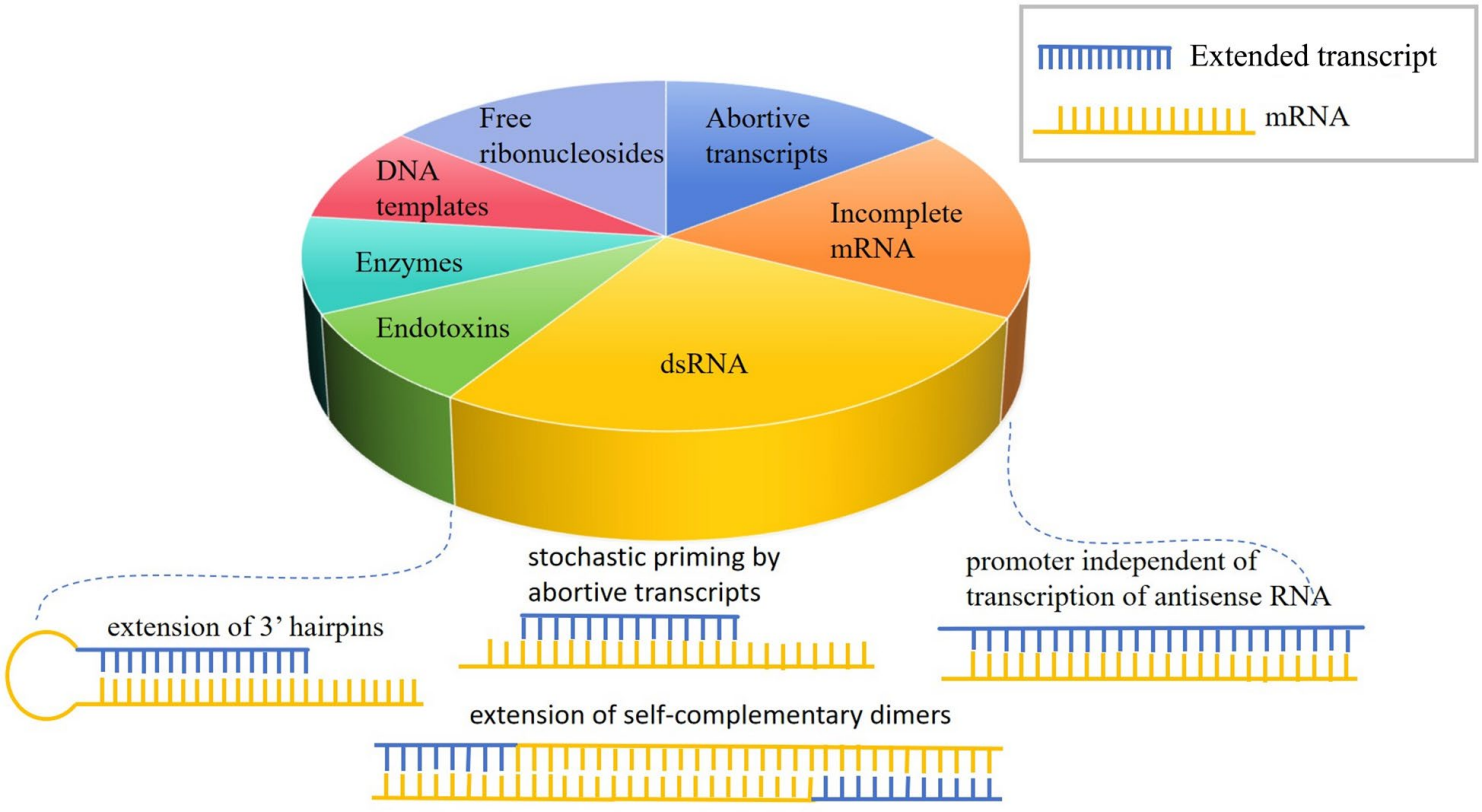
Schematic illustrates the potential contaminants during the in vitro transcription of mRNA

Currently, mRNA is primarily synthesized through IVT using phage-derived T7 RNA polymerase with a DNA template [18]. This well-established method is widely adopted across research laboratories and pharmaceutical industry. However, before IVT-generated mRNA can be used clinically, it requires thorough purification to remove various contaminants present in the reaction mixture. These include residual NTPs, RNA polymerase, DNA templates (and potential endotoxins from plasmid sources), abortive RNA fragments, incomplete transcripts, unincorporated cap analogs, and particularly, dsRNA (Fig. 2) [19]. Among these, dsRNA is especially problematic due to its ability to elicit immune responses, impair mRNA translation, and potentially cause adverse effects in therapeutic applications. Although the exact mechanisms of dsRNA formation remain unclear [20], several pathways have been proposed, including 3' hairpin extension, stochastic priming by abortive transcripts, self-complementary dimer extension, and promoter-independent antisense RNA transcription (Fig. 2) [21, 22]. One proposed mechanism is that T7 RNA polymerase can extend self-primed RNA structures, displaying RNA-dependent RNA polymerase-like activity under certain conditions, enabling it to use previously synthesized RNA as a template for further transcription [23, 24]. If the 3′-end of the runoff transcript exhibits sufficient complementarity (in cis), it can fold back, leading to the extension of the transcript. During transcription initiation, inefficient promoter escape by T7 RNA polymerase can generate short abortive transcripts. These fragments can then anneal with the runoff transcript in a complementary fashion to form dsRNA [23, 25]. In addition, T7 RNA polymerase can also initiate promoter-independent transcription from DNA template ends, generating antisense RNA transcripts that can hybridize with the intended mRNA product [26]. It is important to note that dsRNA does not represent a single, well-defined molecule; rather, it consists of a heterogeneous population of molecules with varying sizes and levels of annealing.

### Immunity triggered by dsRNA

**Fig. 3:**
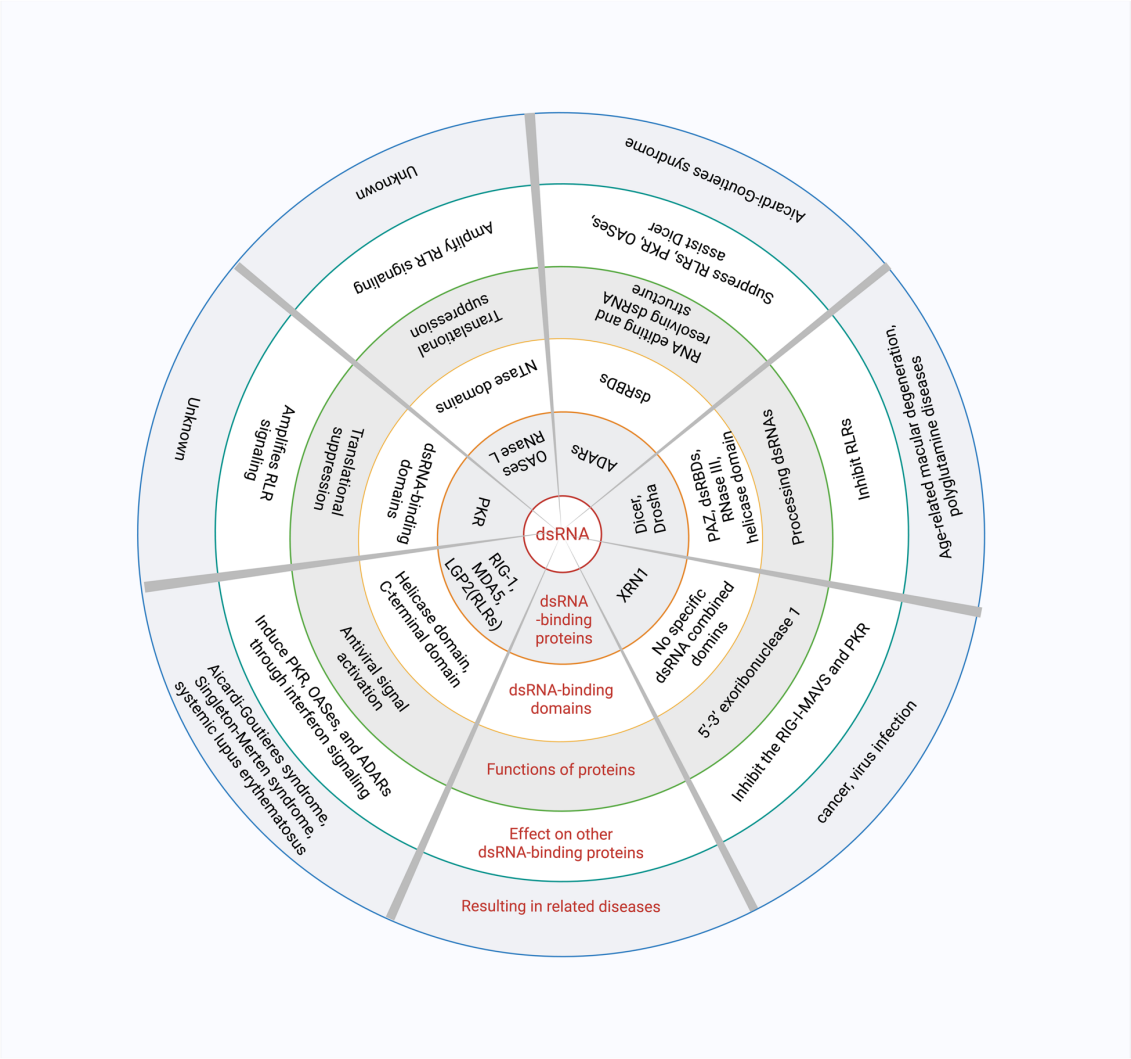
Key dsRNA sensing pathways and their related diseases. Diagram summarizes the key dsRNA sensing pathways and their associated diseases. ADAR: Adenosine Deaminases Acting on RNA (convert adenosine to inosine in dsRNA, RNA editing); dsRBDs: dsRNA Binding Domains; RLHs: RIG-I-like Helicases; LGP2: ATP-dependent RNA Helicase DHX58, a regulator of antiviral signaling and plays a role in innate immunity; MDA5: Melanoma Differentiation-associated Gene 5; OASes: Oligoadenylate Synthases; PKR: Double-stranded RNA-dependent Protein Kinase; RIG-I: Retinoic Acid–Inducible Gene I; RLRs: Retinoic Acid-inducible Gene I-like Receptors; XRN1: 5’-3’ Exoribonuclease 1. The figure was created using BioRender software

**Fig. 4:**
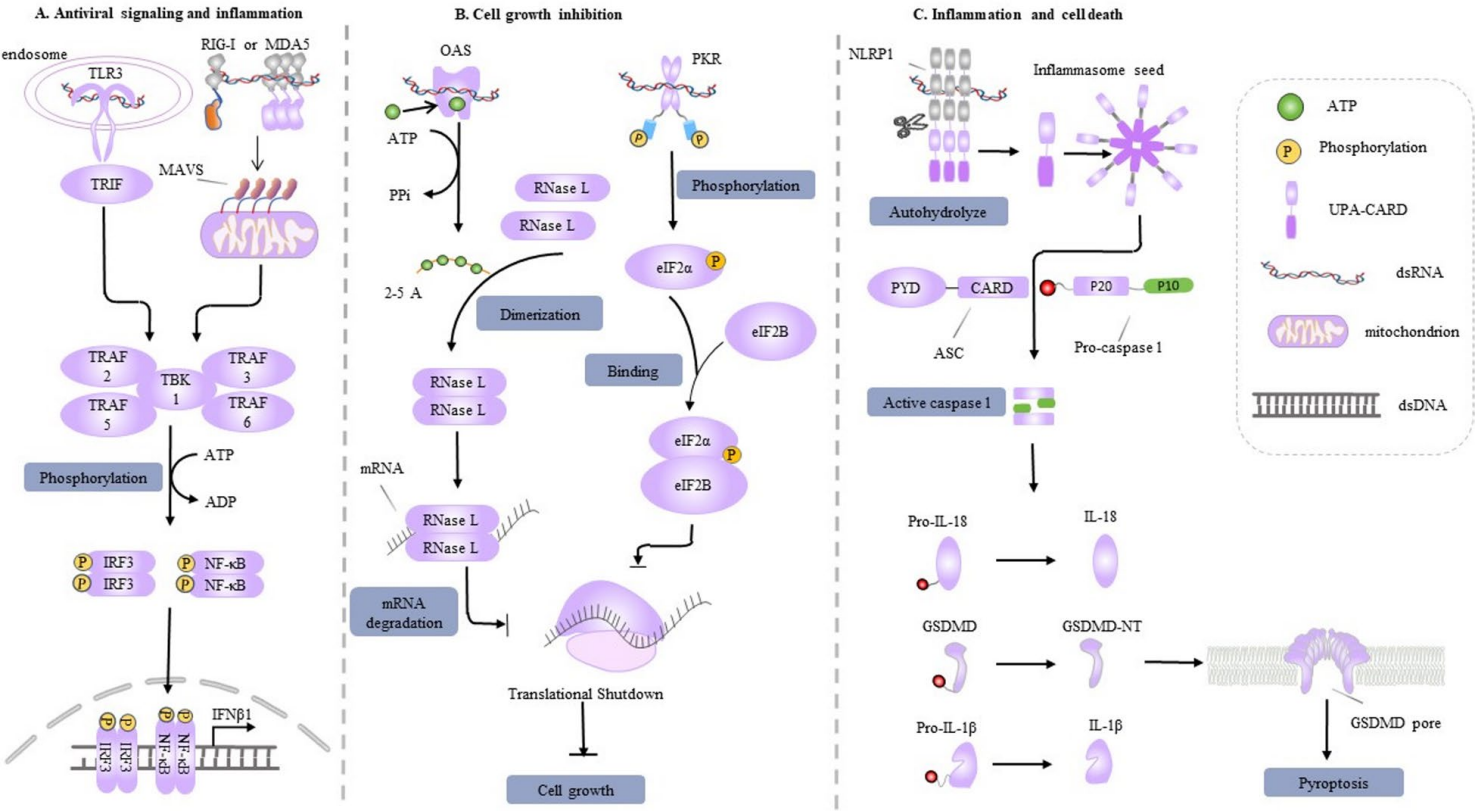
dsRNA sensors and the related signaling pathways. Diagram illustrates three key cellular immune responses triggered by dsRNA. **A**. Antiviral Signaling and Inflammatory Responses: dsRNA activates two key pathways. In the first pathway, dsRNA binds to Toll-like receptor 3 (TLR3) within endosomes, leading to the dimerization of TLR3 and the activation of TRIF. This, in turn, activates downstream kinases TBK1, promoting the phosphorylation of IRF3 and NF-κB. These phosphorylated transcription factors then translocate into the nucleus, where they enhance the transcription and expression of the IFNβ1 gene. The second pathway involves the recognition of dsRNA in the cytoplasm by MDA5 or RIG-I, which then interact with MAVS on the mitochondria. MAVS oligomerization subsequently activates TBK1 and TRAFs, leading to the phosphorylation and nuclear translocation of IRF3 and NF-κB, and ultimately, the upregulation of IFNβ1 gene expression. **B**. Cell Growth Inhibition Pathways: dsRNA can also inhibit cell growth through two primary mechanisms. The first involves the oligoadenylate synthetase (OAS) pathway. When OAS binds to dsRNA, it synthesizes 2'-5' oligoadenylate fragments in the presence of ATP, which activate RNase L. Activated RNase L degrades mRNA, leading to the inhibition of protein translation and, consequently, suppresses the cell growth. The second mechanism is mediated by protein kinase R (PKR). Upon binding to dsRNA, PKR dimerizes and becomes phosphorylated. This activated PKR then phosphorylates the downstream factor eIF2α, inhibiting translation initiation, thereby suppressing cell growth. **C**. Pyroptosis Signaling Pathway. Upon binding to dsRNA, NLRP1 oligomerizes and undergoes proteasome-dependent N-terminal degradation, releasing the UPA-CARD fragment. This fragment forms an inflammasome complex with ASC (Apoptosis-Associated Speck-like protein containing a CARD, also known as PYCARD), which subsequently activates caspase-1. Activated caspase-1 cleaves the precursor proteins of IL-1β and IL-18 and the pore-forming protein gasdermin D (GSDMD). The cleaved GSDMD forms pores in the cell membrane, ultimately leading to pyroptosis

In vertebrates, dsRNA(s) predominantly activate the innate immune response via multiple cellular sensors (Fig. 3). The downstream consequences of dsRNA sensing can be broadly categorized into three major response modes (Fig. 4). The first is the classical antiviral innate immune response, primarily involving retinoic acid-inducible gene I (RIG-I) [27, 28], melanoma differentiation-associated protein 5 (MDA5), and RIG-I-like helicase family member LGP2 [27–32]. Additional sensors, such as RNA-activated protein kinase (PKR) [33, 34] and oligoadenylate synthases (OASes) [35, 36], also contribute to the immune response against dsRNA. Recent studies have identified the human NLRP1 inflammasome as an additional sensor that can respond to long dsRNA and trigger inflammasome activation, which activates caspase-1, resulting in pyroptosis [37]. The immune response triggered by dsRNA by-products mirrors the pathways typically activated by virus-derived dsRNA, leading to immune reaction similar to that induced by viral infections.

For the classical innate immune response, two main pathways are involved: one originates in the endosome, and the other in the cytoplasm. In the endosome, TLR3 recognizes dsRNA, leading to dimerization of TLR3 and activation of downstream adaptor proteins like TRIF. TRIF subsequently recruits TRAF3 and TRAF6, which facilitate the transcriptional activation of NF-κB and IRF3, ultimately inducing interferon (IFN) expression [29, 38]. In the cytoplasm, MDA5 (IFIH1) and RIG-I (DDX58) initiate the response. MDA5 binds to long dsRNA [39], activating mitochondrial antiviral signaling protein (MAVS) on mitochondria, which recruits TRAFs, TBK1, and IRF3 to stimulate IFN signaling. RIG-I detects the 5' triphosphate (5'-ppp) [40], 5'-diphosphate (5'-PP) ends [39, 41] and activates MAVS through a similar pathway [29]. Meanwhile, LGP2 (DHX58), which lacks the N terminal two CARD domains, cannot activate MAVS directly but modulates RNA sensing, particularly by regulating MDA5 filament nucleation and signaling [35].

In the cell growth inhibition pathway, PKR is activated upon binding of dsRNA. This activation leads to the phosphorylation of eukaryotic initiation factor 2 alpha (eIF2α), effectively inhibiting cap-dependent protein translation [35, 42, 43]. Similar to PKR, OAS (2'-5' oligoadenylate synthetase) enzymes are activated by dsRNA and participate in immune defense. The OAS family in humans includes OAS1, OAS2, OAS3, and OASL; of these, OAS1, OAS2, and OAS3 exhibit enzymatic activity. Upon dsRNA binding, these enzymes synthesize 2'-5' phosphodiester- linked oligoadenylates, which activate the endoribonuclease RNase L to degrade RNA as part of the antiviral response [35, 44]. Among these, OAS3 has a stronger affinity for dsRNA compared to OAS1 and OAS2, making it more effective in binding dsRNA and activating downstream RNase L in antiviral immune responses [36, 37]. Another key player in this pathway is the RNA-editing enzyme family ADARs (Adenosine Deaminases Acting on RNA). The ADAR family consists of three primary members: ADAR1, ADAR2, and ADAR3 [45]. Among them, ADAR1 is the most prominent member, as it is ubiquitously expressed and plays a crucial role in regulating basal innate immune activity [37]. As an RNA-editing enzyme, ADAR1 converts adenosine (A) to inosine (I) at specific sites on dsRNA, which can disrupt base pairing to destabilize dsRNA structures and reduce recognition by innate immune sensors [37, 46]. When ADAR1 is deficient (e.g., germline mutation), the RNA sensors are abnormally activated, leading to inappropriate innate immune activation, interferon signaling, and in some contexts cell stress or cell death. This activation can result in autoinflammatory diseases, such as Aicardi-Goutières syndrome [37] and Dyschromatosis Symmetrica Hereditaria [47].

In the third pathway, NLRP1 functions as the key effector. Upon sensing long dsRNA species, human NLRP1 can undergo activation and inflammasome assembly. Following activation, proteasome-dependent degradation of the N-terminal region liberates the UPA-CARD fragment, which nucleates inflammasome assembly. This seed initiates the assembly of the inflammasome complex, leading to the activation of caspase 1. Activated caspase 1 then cleaves the precursors of key inflammatory cytokines, such as IL-1β and IL-18, converting them to their active/mature forms. It also cleaves the pore-forming protein gasdermin D (GSDMD), which inserts into the plasma membrane to form pores, ultimately inducing pyroptosis, a type of inflammation-driven cell death [37].

### Detection of dsRNA

Before therapeutic mRNA can be applied clinically, it is crucial to assess its purity and quantify any residual dsRNA to prevent unintended immune responses and adverse effects. Several methods have been developed to detect dsRNA, including the dot blot assay and ELISA (enzyme-linked immunosorbent assay), both of which utilize J2 (IgG) or K2 (IgM) monoclonal antibodies that specifically recognize dsRNA. These antibody-based techniques are effective at detecting dsRNA duplexes of at least about 40 bp [48–51] and they are widely used to measure residual dsRNA in IVT generated mRNA [22, 49, 52–55]. For example, Luo et al. recently introduced a rapid, sensitive, and user-friendly lateral flow strip assay (LFSA) that uses colloidal gold nanoparticles, enabling visual detection of residual dsRNA in mRNA products within 15 min [56]. Additionally, microfluidic electrophoresis with dynamic double-staining offers a sensitive approach for identifying and quantifying dsRNA during mRNA synthesis, with a detection limit as low as 17.7 pg/μL [57].

Although dsRNA generated during IVT can be sensitively detected and comparatively quantified using J2 (or K2) monoclonal antibody-based assays, universally accepted quantitative thresholds linking specific dsRNA levels to innate immune activation, translational suppression, or regulatory acceptance criteria have not yet been formally established. This is partly because the immunostimulatory effects of dsRNA depend not only on the total amount present, but also on multiple additional factors including dsRNA length, structural heterogeneity, sequence composition, mRNA dose, formulation, route of administration, and the specific cell or tissue targeted. From a practical manufacturing perspective, multiple reports have shown that reducing dsRNA impurities to near or below the detection limit of J2 antibody-based assays markedly improves translational efficiency and reduces innate immune activation in mammalian cells. Accordingly, current industrial practice generally aims to minimize dsRNA to “near-undetectable” levels using purification strategies and sensitive analytical assays. In addition, although regulatory agencies including the FDA and EMA recognize dsRNA as an important process-related impurity and require manufacturers to establish appropriate impurity control strategies and product-specific specifications, there are currently no universally harmonized regulatory thresholds defining acceptable dsRNA levels for therapeutic mRNA products. Instead, acceptable limits are generally determined empirically based on process capability, product characterization, biological activity, innate immune profiling, and preclinical safety data.

### Purification of mRNA

In addition to detection methods, various RNA purification strategies have been developed to remove residual dsRNA and other contaminants from IVT reactions (Table 4). These approaches can be broadly categorized into two main types: (1) post-transcriptional purification, which involves removing impurities after the IVT process, and (2) in-process reduction of dsRNA formation, which focuses on minimizing contaminant generation during transcription. The latter includes the use of engineered or enhanced RNA polymerases, incorporation of chemical reagents, as well as modifying DNA templates to suppress undesired byproducts.

**Table 4:** Comparative analysis of mRNA purification strategies

Purification Method	Separation Principle	dsRNA Removal Efficiency	Scalability	Recovery Yield	Advantages	Limitations
Reversed-Phase HPLC	Hydrophobic interaction between RNA and C18-modified stationary phase	High	Laboratory to industry	Moderate to high	High resolution; compatible with modified nucleosides	High operational cost; solvent toxicity; scale-up complexity
Cellulose-based Chromatography	Selective binding of dsRNA to cellulose under optimized ethanol/salt conditions	High	Laboratory to industry	High (70–80%)	Cost-effective; mild conditions; scalable; rapid processing	Additional downstream polishing required
RNase III	Enzymatic cleavage of double-stranded RNA	Moderate	Laboratory	Variable	Direct dsRNA degradation; simple implementation	Risk of incomplete digestion; potential cleavage of structured mRNA; requires enzyme removal
Oligo(dT) Affinity Chromatography	Hybridization between poly(A) tail and immobilized oligo(dT) ligands	Limited	Laboratory to industry	High (up to ~ 87%)	High specificity for polyadenylated mRNA; solvent-free operation	Insufficient dsRNA removal; requires complementary purification steps
Anion Exchange Chromatography	Electrostatic interaction between negatively charged RNA and positively charged stationary phase	Limited to moderate	Laboratory to industry	80–98%	Scalable; robust and standardized; high recovery rates	Inefficient dsRNA discrimination
Size-Exclusion Chromatography	Separation based on molecular size and pore exclusion	Poor	Laboratory	Moderate	Mild conditions; preserves RNA structural integrity	Low throughput; ineffective for dsRNA removal; limited scalability
Asymmetric Flow Field-Flow Fractionation	Separation based on size, hydrodynamic radius, and diffusion coefficient under cross-flow field	Limited	Laboratory to pilot scale	70–90%	Gentle processing; preserves native RNA conformation	Sample dilution; requires subsequent concentration step
Tangential Flow Filtration	Membrane-based size exclusion under cross-flow filtration	Ineffective	Industry	> 95%	Cost-effective; scalable; suitable for continuous processing	Cannot distinguish dsRNA from mRNA of similar size; membrane fouling issues
Selective Precipitation	Differential solubility under high salt conditions	Poor	Laboratory	~ 80%	Simple and cost-effective	Incomplete dsRNA removal; salt residue concerns
Silica-based Purification	RNA adsorption to silica in high-salt buffers	Poor	Laboratory	Moderate	Widely available commercial kits; standardized protocols	Ineffective dsRNA removal; not suitable for large-scale manufacturing

### mRNA purification and dsRNA removal

mRNA is typically synthesized through IVT, and its purification is essential for both laboratory-scale and industrial applications. The commonly used purification methods include reversed-phase high-performance liquid chromatography (RP-HPLC), affinity chromatography using oligo (dT), cellulose-based purification, anion exchange chromatography (AEX), and tangential flow filtration (TFF, primarily used for concentration, buffer exchange, and removal of low-molecular-weight impurities). Several of these methods are scalable and compatible with high-throughput or industrial processing, although their degree of continuity and manufacturing suitability varies by platform. Chromatographic methods are attractive because they can provide selective, scalable separations and are compatible with standardized manufacturing workflows [58, 59]. They purify mRNA by leveraging differential interactions between the mRNA and the stationary or mobile phase, achieving precise separation from contaminants. TFF is widely used in scalable bioprocessing, whereas asymmetric flow field-flow fractionation (AF4) is a gentle size-based separation method that has also been explored for RNA purification and characterization. There are also several purification methods that are generally used more selectively depending on the scale and purification objective. These include techniques such as size exclusion chromatography, selective precipitation, and silica-based purification methods, which are also summarized in this review.

### dsRNA removal: reversed-phase HPLC (RP-HPLC)

**Fig. 5:**
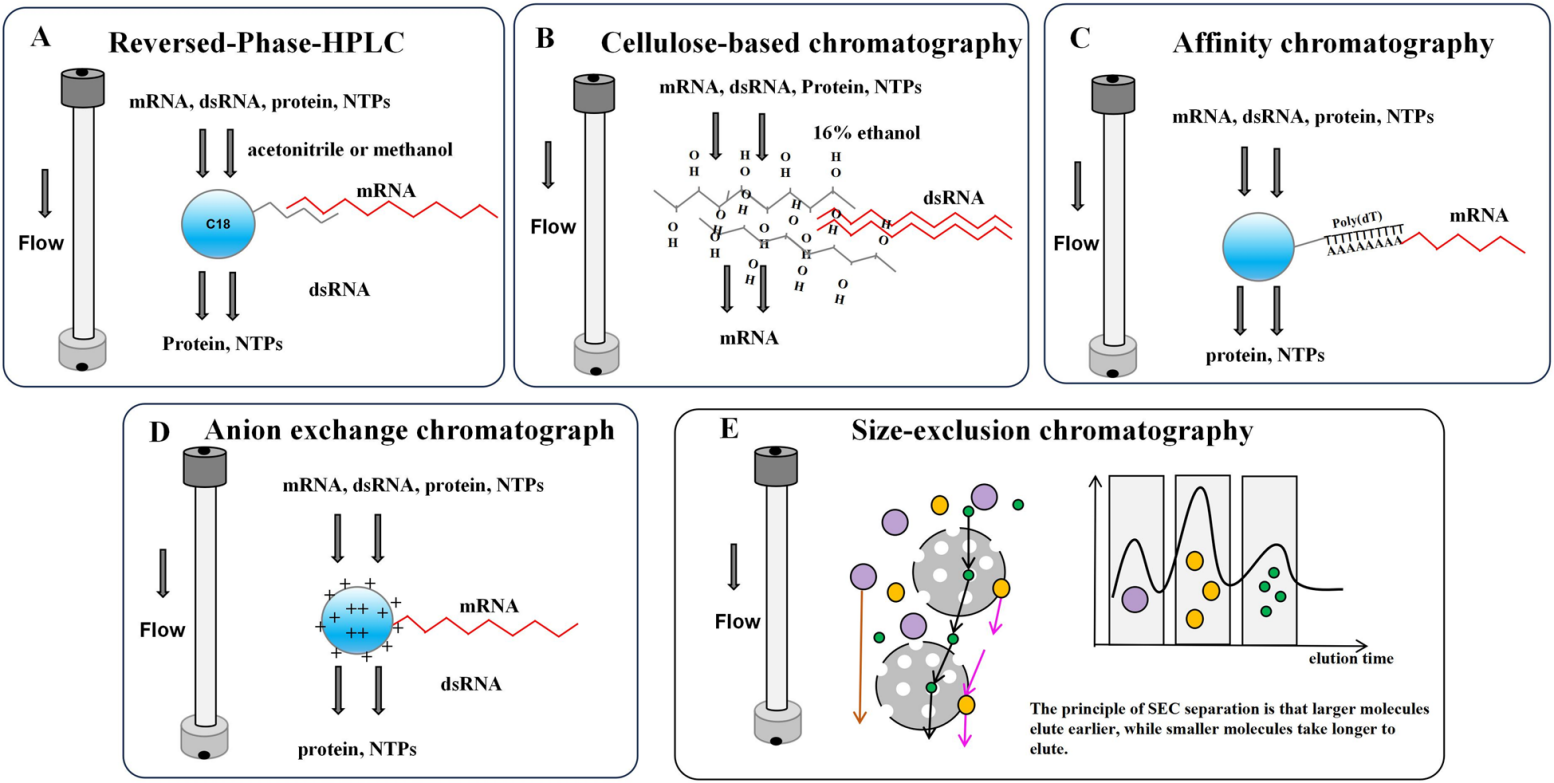
Schematic diagram illustrates five chromatographic methods for purifying mRNA. **A**. Reversed-phase high-performance liquid chromatography. **B**. Affinity chromatography. **C**. Cellulose -based purification. **D**. Ion-exchange chromatography. **E**. Size-exclusion chromatography

RP-HPLC (Fig. 5A) has been widely applied in mRNA purification [60, 61] and dsRNA removal. mRNA is a long-chain molecule composed of nucleotide residues with negative charges, which also exhibit some degree of hydrophobicity. In the presence of ion-pairing reagents, mRNA interacts with hydrophobic C18 stationary phases, resulting in retention and chromatographic separation. By carefully selecting an appropriate mobile phase, such as acetonitrile or methanol, various components can be gradually eluted from the stationary phase, facilitating effective purification [59]. For example, octadecyl-based RNA-RP1 columns operated with triethylammonium acetate-containing mobile phases have been used to effectively separate single-stranded RNA from double-stranded RNA [62]. The removal of dsRNA is often important for maximizing translation and minimizing innate immune activation. In one study, octadecyl-based chromatographic columns (e.g., RNASep™ Prep) for mRNA purification led to a 10- to 1000-fold increase in target protein expression upon transfection into primary cells, along with significantly reduced induction of the unintended IFN and inflammatory factors [48]. A detailed protocol for purifying long-chain RNA using traditional HPLC, including loading, purification, recovery, and detection steps, which can be scaled up for large-scale mRNA production has been outlined by Weissman et al. [63].

Besides the regular RP-HPLC, some groups have developed cap analogs bearing hydrophobic handles that enable chromatographic separation of capped from uncapped transcripts by RP-HPLC. This selective binding enables the efficient removal of uncapped mRNA as well as the abortive transcripts and the double-stranded RNA, thereby significantly enhancing the efficiency and capacity of mRNA purification [64]. Warminski et al. developed AvantCap, a trinucleotide FTO-resistant N6-benzyl analogue of the ^m6^A_m_-cap–m^7^Gppp^Bn6^A_m_pG. When incorporated into mRNA, AvantCap significantly improves RP-HPLC purification efficiency, reduces dsRNA content, and enhances translation efficiency [65]. In addition, Masahito Inagaki et al. introduced hydrophobic photocaged tag-modified cap analogs (e.g., PureCap analogs), enabling the generation of fully capped mRNA with 100% efficiency through two rounds of RP-HPLC purification [64].

Beyond refining the 5' cap structure of RNA, optimizing experimental protocols and methodologies offers another effective strategy. For instance, optimization of ion-pair RP-HPLC conditions further enhances contaminant removal, yielding purity levels suitable for crystallographic applications [66]. Andreja Krušič et al. developed a stepwise elution method that, by optimizing the ion-pairing RP-HPLC process, enables the production of high-quality mRNA and reduces the activation of innate immune pathways in cell-based assays [67]. In addition, the pore size of the separation medium has a significant impact on the resolution of RP-HPLC, indirectly influencing RNA separation and purification. Makoto Ozaki et al. studied octadecyl-based RNA-RP1 columns with different pore sizes and found that super wide pore (> 30 nm) columns improved chromatographic resolution and analytical separation of single-stranded RNA (ssRNA) of various lengths [68]. Moreover, RP-HPLC is broadly compatible with many modified RNA constructs, underscoring its potential for mRNA vaccine and therapeutic drug development [69, 70].

Despite its high efficacy, RP-HPLC has certain limitations, including the high cost associated with large-scale applications and the use of toxic solvents such as acetonitrile, which must be thoroughly removed following purification [59, 71].

### dsRNA removal: cellulose-based purification (CBP)

Cellulose-based chromatography (Fig. 5B) is an effective method for dsRNA removal [51, 72–75]. As a polysaccharide, cellulose can form strong interactions with dsRNA under optimal buffer conditions (e.g., 16% ethanol and 125 mM NaCl), efficiently removing it from the IVT product, while the desired mRNA remains in solution and flows directly through the column [22, 72–74, 76, 77]. This method has been shown to remove approximately 90% or more of detectable dsRNA from reaction products, with mRNA recovery rates of 65–90% [4, 5, 72, 73, 78–81]. Efficient removal of dsRNA prevents activation of PKR signaling, thereby enhancing the translational efficiency of the target mRNA.

Recently, Yuan et al. utilized natural wood-derived macroporous cellulose, which efficiently removed 98% of dsRNA from mRNA products within 5 min, significantly reducing the in vivo immunogenic response. Wood-derived macroporous cellulose can also be adapted to create chromatography columns of varying sizes, enabling large-scale mRNA purification when integrated with high-performance liquid chromatography [75]. Zhang et al. developed a method that combines RNase R with wood-derived macroporous cellulose for circRNA purification, achieving a purity level of up to 95%. This approach demonstrated a recovery rate exceeding 70%, while effectively minimizing unintended immune activation and reducing the expression of immunogenic factors [82]. Moreover, sa-mRNA (Self amplifying mRNA, Box1) purified using cellulose-based techniques produces protein expression levels that are significantly higher than those obtained through the commonly used, silica-based RNA purification [83]. Similarly, Cui et al. demonstrated that cellulose-based purification significantly reduces dsRNA levels in sa-mRNA compared to the silica-based purification [84].

Compared to other methods such as RP-HPLC, the cellulose-based method offers simplicity and cost-effectiveness for dsRNA removal, making it attractive for scale-up production and process simplification [67]. Additionally, it operates under relatively mild conditions and avoids the use of toxic solvents like acetonitrile. However, its specificity is relatively limited, as it selectively binds and removes dsRNA but does not eliminate other impurities such as template plasmid DNA, T7 RNA polymerase, unincorporated NTPs, and truncated or uncapped RNAs. These impurities, along with the desired mRNA, remain in solution and flow through the column [72]. Combining cellulose-based purification with additional purification methods in subsequent steps is often required to further improve the overall purity of the final mRNA product.

### dsRNA removal: dsRNA-degrading enzymes

The use of ribonuclease III (RNase III) enzymes has emerged as a novel strategy to remove residual dsRNA from IVT reaction products. RNase III preferentially recognizes and cleaves dsRNA structures, thereby reducing dsRNA contaminants in IVT-derived mRNA preparations. Following enzymatic digestion, additional purification steps are typically performed to remove the enzyme and digestion products before downstream use [71]. This approach has been shown to improve CAR expression and T-cell functionality while reducing the unwanted activation of the innate immune response [85].

**Fig. 6:**
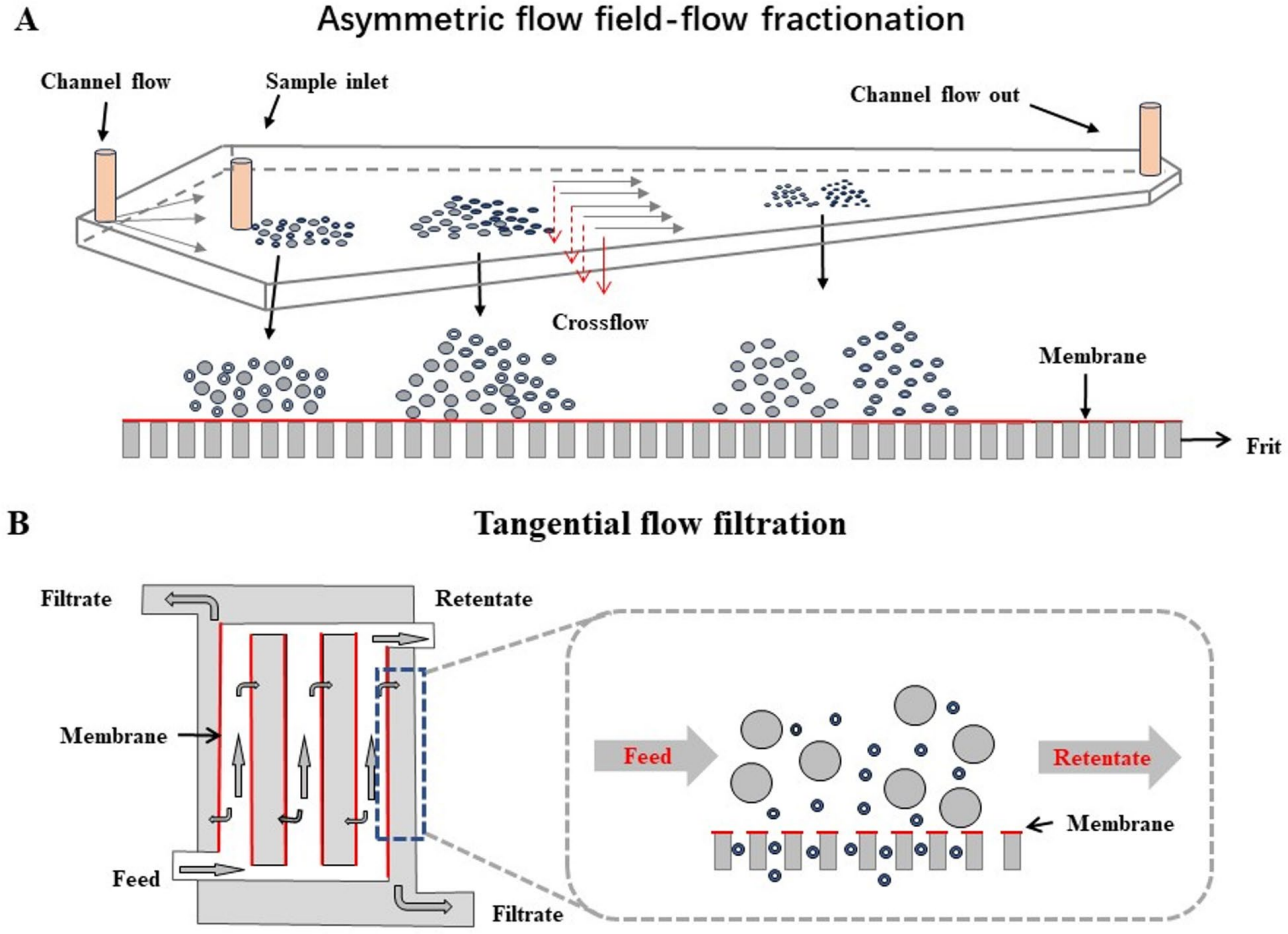
Schematic diagram illustrates two flow separation methods for purifying mRNA. A. Asymmetric flow field-flow fractionation. B. Tangential flow filtration

While the application of RNase III facilitates the selective degradation of dsRNA by-products, this approach has certain limitations [71]. Incomplete RNase III digestion of dsRNA may result in the production of smaller dsRNA fragments, which may still activate immune responses [26, 39, 72]. Because RNase III recognizes dsRNA structure rather than specific sequences, highly structured regions within the target RNA could theoretically be susceptible to cleavage if reaction conditions are not carefully optimized [26]. Another limitation is that RNase III, being an exogenous enzyme, requires thorough removal through additional purification steps [26, 72]. In addition, RNase III treatment introduces an extra enzymatic processing step and generates cleavage products that must be removed during downstream purification, increasing process complexity and manufacturing costs.

### General mRNA purification: affinity chromatography (AC)

Affinity chromatography (Fig. 5C) was used for mRNA purification as early as the 1970s [86]. The widely used and successful method to date is oligo (dT) based affinity chromatography [6]. Functionalization of the stationary phase with immobilized oligo (dT) ligands enables the specific capture of poly(A) tailed mRNA molecules [87]. Because oligo (dT) chromatography captures poly(A)-containing transcripts based on sequence complementarity rather than RNA secondary structure, residual dsRNA impurities may remain after purification [67, 71, 81]. Compared with RP-HPLC, oligo(dT)-based affinity chromatography typically avoids the use of organic mobile phases such as acetonitrile, simplifying solvent handling and reducing solvent consumption during purification [88, 89].

Dewar et al. compared three commercially available oligo dT affinity columns and found that Fibro™ exhibited 2–4 times higher dynamic binding capacity for mRNA compared to CIMmultus^®^ and 2–13 times higher than POROS™ [87, 90, 91]. Tan et al. demonstrated that Oligo dT resin with an optimal pore size of about 350 nm had the highest dynamic binding capacity for the tested mRNA molecules, with polymer grafting enhancing the binding capacity by approximately 24–55% across various mRNA lengths [92]. To meet the growing demand for large-scale clinical mRNA production, Mencin et al. utilized an 800 mL CIMmultus Oligo dT column to purify eGFP mRNA, achieving a loading capacity of 1.5 g with a recovery rate of 87% [93]. Similarly, Boman et al. developed an optimized IVT process that, together with downstream affinity chromatography purification, produced mRNA preparations with low or undetectable dsRNA levels under the tested conditions. This advanced method enhanced the efficiency of IVT reaction component utilization by 44%, leading to an increased mRNA yield of 24.9 ± 1.5 g/L while significantly reducing production costs [94].

### General mRNA purification: anion exchange chromatograph (AEX)

Anion exchange chromatography (Fig. 5D) is a highly effective technique for the large-scale purification of mRNA, exploiting differences in charge between mRNA molecules and impurities for precise separation [6, 95]. In AEX, the negatively charged mRNA interacts with the positively charged groups on the stationary phase surface [96, 97]. By adjusting the salt concentration in the mobile phase (e.g., adding NaCl), the interaction between mRNA and the stationary phase can be modulated. Higher salt concentrations weaken the electrostatic binding, leading to the elution of the mRNA. Polona Megušar et al. achieved mRNA yields of up to 98% using an enhanced PrimaS column, with stability maintained for 28 days at room temperature [98]. Similarly, Rok Miklavčič et al. developed a weak AEX chromatographic material that eluted mRNA at neutral pH and room temperature, with recovery rates exceeding 80% and mRNA stability lasting up to 34 days [99]. Emma Welbourne et al. optimized an AEX HPLC method, enabling the separation of impurities from IVT products in just 6 min, significantly enhancing the mRNA production efficiency [100].

While AEX is widely used for mRNA purification, some AEX based methods require denaturing conditions, such as elevated temperatures (e.g., 50–65 °C) and chaotropic agents (e.g., urea, guanidinium chloride) [59], which must be thoroughly removed prior to downstream applications. The AEX method may not fully remove dsRNA on its own and thus often needs to be combined with additional purification methods [67, 71, 101, 102]. In addition, elution often requires relatively high salt concentrations, necessitating subsequent desalting or buffer-exchange steps prior to formulation.

### General mRNA purification: size-exclusion chromatography (SEC)

Size-exclusion chromatography (Fig. 5E) separates molecules based on their molecular size [6, 103]. This method can separate the unreacted nucleotides, and short transcripts from IVT-derived large mRNA [104], but is generally not effective at removing dsRNA due to their similar molecular size. The SEC column is packed with porous spherical fillers, such as cross-linked dextran, polyacrylamide, silica gel, or agarose-based materials, each designed with a specific pore size range [105, 106]. As the sample solution enters the column, molecules of different sizes interact with the filler pores to varying degrees. Smaller molecules penetrate the pores more extensively, increasing their retention time, while larger molecules experience less penetration and elute more quickly [103]. This size-dependent interaction results in distinct elution times, with smaller molecules eluting later than larger ones [106–108]. D'Atri et al. reported that, among the SEC columns evaluated, ~ 1000 Å pore-size columns provided the best analytical performance for mRNAs in approximately the 0.5–5 kb range [109]. Similarly, De Vos et al. investigated how different pore sizes of SEC resins affect the separation efficiency and structural integrity of mRNAs of varying lengths. Columns packed with 300 Å pore media are recommended for analyzing mRNA fragments shorter than 0.5 kb. For mRNAs between 0.5 and 5 kb, 1000 Å resins are recommended, while 2000 Å resins are more suited for self-amplifying RNA (saRNA) longer than 5 kb [110]. A recent study on pore sizes (550–1000 Å) provides more detailed recommendations for the analytical characterization of mRNAs of different lengths (1000–4500 nt) [111].

The limitation of SEC for RNA purification is its relatively low loading capacity and productivity, which restricts its utility for large-scale industrial application. In addition, SEC often results in sample dilution during elution, necessitating subsequent concentration steps. SEC is generally not the preferred method for dsRNA removal because dsRNA often overlaps with product RNA in size.

### General mRNA purification: asymmetric flow field-flow fractionation (AF4)

Asymmetric flow field-flow fractionation (Fig. 6A) is a gentle and efficient technique for separating and purifying macromolecules based on their size, hydrodynamic properties and diffusion coefficients [112, 113]. This separation process utilizes cross-flow within a narrow, flat, and elongated trapezoidal channel. The AF4 channel consists of a solid upper wall and a semipermeable lower accumulation wall, in which a microporous membrane is supported by a porous frit or support plate. Macromolecules are retained within the AF4 channel by the semipermeable accumulation wall that allows solvent to pass through while retaining analytes above the membrane cutoff [114–118]. By modulating cross-flow rate and channel flow conditions, AF4 enables efficient size-based separation of RNA molecules [116]. Katri et al. optimized AF4 conditions to successfully separate ssRNA from dsRNA, achieving a recovery rate of 70–90%, which remained consistent across RNA molecules of varying lengths [116].

**Fig. 7:**
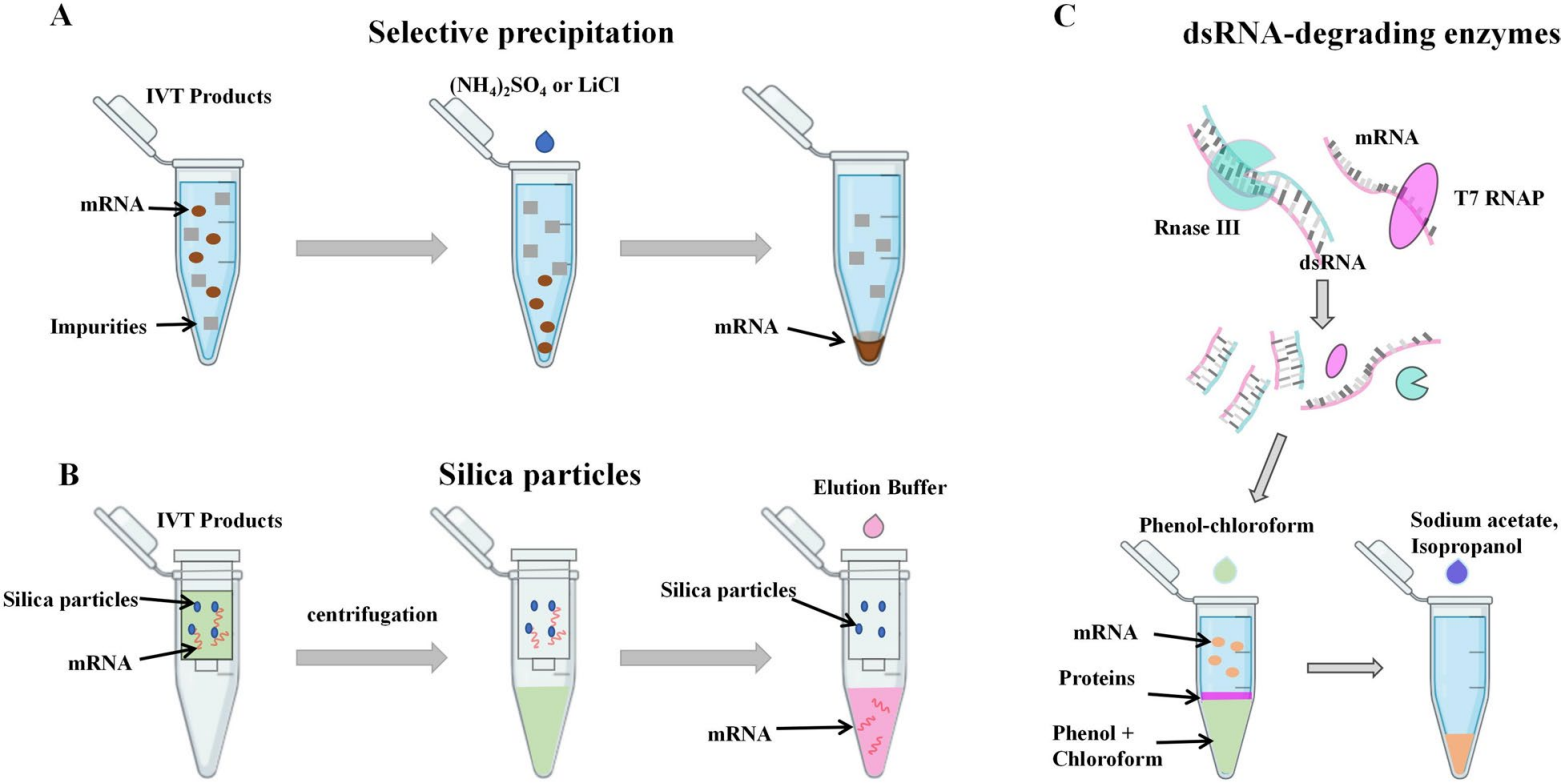
Schematic diagram illustrates three less common mRNA purification methods. A. Selective precipitation. B. Silica particles. C. dsRNA-degrading enzymes

AF4 purification is a gentle and non-denaturing RNA purification method. Notably, some chromatographic and electrophoretic based RNA purification methods include a denaturation step to disrupt secondary and tertiary structures. However, this denaturation process may sometimes hinder the RNA from fully refolding into its native conformation. In contrast, AF4 purification occurs within an open channel, where the absence of a packed stationary phase, reduced analyte-surface interactions, and relatively low shear stress provide mild processing conditions that help preserve native RNA structures [113, 114]. By optimizing AF4 parameters, the product yield can exceed 70% [116]. The drawback of AF4 technique is that it dilutes the sample during purification, requiring subsequent concentration to meet usage requirements. Additional challenges include analyte adsorption to the accumulation membrane, and relatively low throughput compared with industrial chromatographic purification platforms. AF4 method development can also be challenging because separation performance is highly dependent on membrane selection, carrier composition, and cross-flow programming.

### General mRNA purification: tangential flow filtration (TFF)

Tangential flow filtration (Fig. 6B) (also known as crossflow filtration) is a size-based membrane filtration technology used for concentration and buffer exchange of biomolecules [59]. This technique relies on two perpendicular liquid flows: a tangential crossflow along the membrane surface combined with a pressure-driven permeate flux across the membrane. As molecules travel at different speeds within these flows, target molecules are effectively separated from impurities [119]. The production method used for SARS-CoV-2 mRNA involves TFF, where the vaccine mRNA is purified by oligo-dT affinity chromatography and then buffer exchanged by TFF into sodium acetate [120]. Filtering the IVT RNA mixture through a membrane with a molecular weight cutoff (e.g., 100 kDa) and a tenfold filtration volume effectively removes small molecules such as NTPs, salts, and polycations (e.g., spermidine), while retaining mRNA and reduces the protein-to-RNA ratio by a factor of 50 [121]. A recent study showed that adding a vibration module to single-pass TFF enabled a tenfold concentration of mRNA, a nearly tenfold reduction in nucleoside triphosphates, approximately 60% protein removal, and the preservation of mRNA integrity [122].

TFF is well-suited for large-scale industrial production due to its relatively low operating costs, scalability, and mild purification conditions. However, it is ineffective for dsRNA removal as the dsRNA often has similar size to the target mRNA and cannot be separated through membrane filtration. Other drawbacks of TFF purification include membrane fouling and concentration polarization (Box 1), which are frequently cited as significant limitations [123]. Membrane fouling occurs when particles, proteins, cell debris, or other contaminants accumulate on or within the membrane, leading to reduced flux, altered retention rates, and decreased filtration efficiency. Concentration polarization arises when selective membrane transfer creates steep concentration gradients at the membrane-solution interface, further affecting filtration performance [124]. To mitigate concentration polarization and membrane fouling, TFF often operated with high flow rates. However, these high flow rates can compromise purification efficiency and recovery rates, necessitating multiple cycles to achieve the desired outcomes. Ehsan Nourafkan et al. evaluated membrane fouling model using experimental data from TFF, providing a detailed characterization of mRNA adsorption on the membrane surface. Under optimized conditions, the study achieved high-purity separation of mRNA from unreacted NTPs with recovery greater than 70% and no detectable mRNA degradation [125]. In addition, the recent development of Single Pass Tangential Flow Filtration (SPTFF) also provide an improved strategy through concentrating the product in a single pass through a series of membranes, reducing processing volume and eliminating the need for recirculation steps [126].

**Table:** **Box 1** Definition of Terms

**Membrane fouling:** Membrane fouling refers to the phenomenon where particles, proteins, cell debris, or other contaminants accumulate or block the membrane surface or pores, leading to a decline in filtration performance, such as reduced flux, changes in retention rate, and decreased filtration efficiency	
**Concentration polarization:** Concentration polarization occurs at the interface between the membrane and the solution in TFF. It is caused by the accumulation of product molecules on the membrane surface due to solvent flow through the membrane, resulting in a concentration gradient that cannot be dissipated in a timely manner	
**Chaotropic agents:** Chaotropic agents are a class of chemical substances that disrupt the non-covalent interactions (such as hydrophobic interactions, ionic bonds and hydrogen bonds) within biological macromolecules (such as proteins, DNA, and RNA), leading to their structural disintegration or denaturation	
**Sa-mRNA:** Sa-mRNA (self-amplifying mRNA) is an mRNA molecule engineered to replicate itself. It retains the genes responsible for the replication machinery of Alphavirus RNA, while the genes encoding viral structural proteins are replaced with the gene encoding the target protein. Once delivered into the cytoplasm, it can continuously and abundantly translate the target protein [170]	

### General mRNA purification: selective precipitation

Selective precipitation (Fig. 7A) is a traditional technique for RNA recovery and purification. This method relies on the differential solubility of molecules in specific solutions to achieve the separation of mRNA. The fundamental principle behind this technique is based on the intrinsic properties of RNA: as a negatively charged and highly polar molecule, RNA readily dissolves in water. To precipitate RNA from an aqueous solution, it is necessary to neutralize the negative charges on its backbone. This is typically achieved by adding monovalent cations in the form of salts. Among the commonly used precipitation salts in classical RNA protocols are sodium acetate, ammonium acetate, and lithium chloride [127], whereas ammonium sulfate has more recently been explored for IVT mRNA purification under room-temperature conditions. Feng et al. reported that ammonium sulfate precipitation under optimized room-temperature conditions, including 2 M ammonium sulfate at pH 7.0, enabled rapid and high-recovery isolation of IVT mRNA [128]. However, this approach has limited selectivity for removing abortive RNA and dsRNA contaminants [6]. Lithium chloride precipitation, using a 2–2.5 M solution, preferentially precipitates high-molecular-weight RNA including mRNA and offers improved RNA recovery [129]. A practical limitation of salt-based precipitation methods is that residual salts must be adequately removed before downstream use [128]. Additionally, some classical precipitation workflows involve chilled incubation (e.g., in − 20 °C), which can increase processing cost and infrastructure requirements [127].

### General mRNA purification: silica particles

Silica-based purification (Fig. 7B) is a conventional nucleic-acid isolation method that relies on nucleic-acid adsorption to silica under chaotropic high-salt conditions [130, 131]. In mRNA workflows, silica-based formats are more commonly used in laboratory-scale purification and sample preparation than as primary large-scale polishing steps. The underlying principle relies on the presence of high-salt buffers (e.g., GuHCl), which disrupt the hydration shell surrounding nucleic acids [131, 132]. Under chaotropic high-salt conditions, disruption of hydration shells promotes adsorption of nucleic acids onto the silica surface. Subsequent washing steps with appropriate buffers (e.g., ethanol-containing solutions) effectively remove contaminants/impurities, including proteins, salt ions, and NTPs, while retaining mRNA on the silica matrix. Finally, under low-salt or salt-free conditions (such as low-ionic-strength Tris–EDTA buffer or RNase-free water), the hydrogen bonding and electrostatic interactions between mRNA and silica particles are weakened, allowing for the efficient elution and recovery of purified mRNA [132].

Despite its robust performance in RNA purification and in removing salt ions and dNTPs, the silica particle/matrix-based method is generally ineffective for dsRNA removal. Recently, a few studies have developed new approaches using modified silica particles (such as mesoporous silica) for dsRNA removal. For example, in a study conducted by Cho et al., mesoporous silica particles were shown to be effective RNA adsorbents for purifying IVT products. In that study, they achieved approximately an 80% reduction in residual dsRNA [130].

## Reducing dsRNA formation during IVT reaction

The methods for removing dsRNA discussed earlier are typically applied after mRNA synthesis. However, these approaches are often time-consuming, labor-intensive, and costly, and they also often result in significant mRNA loss during purification. Preventing or minimizing dsRNA formation during mRNA synthesis may reduce the extent of downstream purification required, thereby increasing mRNA yield, lowering costs, and reducing the time required for RNA manufacture. This presents a potential effective alternative to post-transcriptional purification [133].

### Reducing dsRNA formation by using improved RNA polymerase

Researchers have engineered various RNA polymerase mutants to reduce or eliminate dsRNA production during IVT. One of the examples is VSW-3 RNA polymerase identified from psychrophilic phage. This RNA polymerase shows reduced propensity for RNA-dependent extension or self-priming compared to T7 RNA polymerase; and unlike T7 RNA polymerase, this enzyme is less likely to use RNA transcripts from the IVT reaction as templates to extend mRNA at the 3' end. This unique property reduces the synthesis of extended RNA sequences and reduces the formation of hairpin-like dsRNA at the 3' end. Furthermore, without RdRp activity, VSW-3 polymerase cannot use abortive transcripts to generate separate sequences partially complementary to the mRNA, further hindering the formation of complementary dsRNA [134]. Xia et al. demonstrated that VSW-3 RNA polymerase (and its engineered mutants) produced minimal abortive transcripts and dsRNA at temperatures ranging from 4 to 25 °C. Notably, with optimal buffer and the same concentrations of enzyme and substrates, the maximum yields of VSW-3 and T7 RNA polymerase were comparable [52, 134]. Importantly, Wang et al. demonstrated that mRNA synthesized with VSW-3 RNA polymerase had significantly lower levels of dsRNA compared to mRNA produced by wild-type T7 RNA polymerase [134]. Another example is the use of thermostable RNA polymerases. Wu et al. and Nagaraj et al. demonstrated that commercially available thermostable RNA polymerases [TsT7-1 and TsT7-2, both remain active at elevated temperatures (50 °C)] produce mRNA yields comparable to wild-type T7 RNA polymerase but with significantly reduced immunogenicity [133, 135]. The key mechanism underlying this reduced immune activation lies in the transcription conditions: at the standard temperature of 37 °C, run-off transcripts can rebind the polymerase and self-prime at their 3′ ends, leading to the formation of long 3′-extended duplexes. Performing IVT at higher temperatures with thermostable RNA polymerases prevents this rebinding and self-priming, thereby reducing the formation of dsRNA by-products.

In addition to developing and testing new RNA polymerases, researchers have also made modifications to the protein sequences of the T7 RNA polymerase using in vitro protein evolution techniques. For example, Wu et al. developed a single-mutant T7 RNA polymerase, S43Y, which significantly attenuates the RNA dependent RNA polymerase activity without compromising the enzyme's DNA-dependent RNA polymerase activity [136]. Similarly, Yu et al. engineered other single-mutant variants, such as G47W, which enhanced the expression efficiency of the IVT-synthesized mRNA while reducing immunogenicity [137]. The G47W mutation diminishes non-promoter-dependent initiation events at DNA ends, preventing antisense RNA synthesis and minimizing the formation of full-length double-stranded RNA. Building on this knowledge, Dousis et al. developed multi-site T7 RNA polymerase mutants. One of their notable developments is the double-mutant T7 RNA polymerase, G47A/884G, which significantly reduces dsRNA production during the synthesis of mRNAs of varying lengths. This mutant also improves the 3′ homogeneity of mRNA and reduces innate immune responses [138]. Additionally, Tang et al. engineered a triple-mutant T7 RNA polymerase, named Mut17 (A70Q/F162S/K180E, generated via DNA shuffling), which significantly reduced dsRNA production under various conditions, while maintaining the quality and quantity of the resulting full-length cap-mRNA. Further cellular experiments showed that this engineered mutant RNA polymerase significantly decreased the unintended immune stimulation in cells [139]. Miller et al. developed a novel RNA polymerase mutant, T7-68, which efficiently utilizes lower concentrations of m^7^G for co-transcriptional capping, achieving over 95% capping efficiency while significantly reducing dsRNA byproducts during the reaction. When transfected into HeLa cells, it notably increased target protein expression and reduced immunogenicity [140].

### Chaotropic agents inhibit dsRNA formation during the IVT reaction

Chaotropic agents (Box 1), such as urea and formamide, can disrupt undesired nucleotide base hybridization, thereby reduce the formation of unwanted dsRNA while preserving mRNA yield. Piao et al. demonstrated that adding 1 M urea or 1.6 M formamide to the standard IVT reaction reduced dsRNA by 80% without affecting the yield of mRNA [54]. This approach led to lower immunogenicity and improved protein expression efficiency. Similarly, Combes Francis et al. showed that incorporating urea into the IVT reaction also reduced dsRNA byproducts and decreased IFN expression in macrophages [141]. Collectively, these findings highlight the potential of chaotropic agents as effective additives for minimizing dsRNA formation during IVT. As most chaotropic agents are small molecules, they can be readily removed after the IVT reaction through dialysis or desalting columns.

### Enhancing local enzyme concentration to reduce dsRNA formation during the IVT process

Cavac et al. demonstrated that coupling T7 RNA polymerase and a DNA template to magnetic beads enhances local enzyme concentration, thereby improving transcription efficiency. Their study showed that performing IVT under high-salt conditions (0.3 M NaCl) significantly reduces dsRNA by-products while also increasing mRNA yield [142].

### Modifying DNA templates or RNA to reduce dsRNA formation during the IVT reactions

Researchers have also explored various strategies for reducing dsRNA content in transcription products, moving beyond engineered RNA polymerase mutations. These approaches include targeted modifications to both DNA templates and RNA products. In terms of DNA template design, Sari et al. demonstrated that incorporating an A/T-rich sequence downstream of the T7 promoter could reduce dsRNA levels by 30%, which subsequently improved target protein expression in cells [143]. In a related study, MalagodaPathiranage et al. introduced deoxyuridine (dU) at the -4 position of the non-coding DNA strand in the promoter region, followed by USER^®^II enzyme cleavage. This technique enhances the RNA polymerase promoter binding, and significantly reduces dsRNA formation, especially under high-salt conditions [144]. Another strategy involves incorporating a short "capture" DNA oligonucleotide complementary to the RNA’s 3'end. This binding prevents the RNA from folding back on itself, effectively inhibiting self-primed extension. By eliminating primer-extended byproducts, this method significantly enhances the purity of the RNA product and yields substantially higher amounts of target mRNA [145]. In addition to modifying and optimizing DNA template sequences, recent studies suggest that the DNA template purity also plays an important role in dsRNA formation. For example, Martínez et al. reported that plasmid impurities present during the IVT reaction can act as unintended templates, resulting in the synthesis of fragmented ssRNA that anneals with the primary transcripts to form dsRNA. Thus, the purity and integrity of linear DNA templates are crucial for minimizing dsRNA formation, underscoring the importance of thorough DNA template purification [20].

Beyond DNA template modifications, optimizing the NTP composition in the IVT reaction is also crucial for DNA templates containing poly(A) coding sequences. Specifically, maintaining low steady-state concentrations of UTP or modified UTP (m^1^ψTP), particularly in combination with GTP feeding, can reduce dsRNA formation during IVT. This strategy not only reduces dsRNA byproducts but also preserves mRNA yield and integrity, ultimately improving protein expression efficiency [53].

## Discussion

Advances in mRNA technology have revolutionized modern therapeutics, opening new avenues for vaccine development and the treatment of a wide array of diseases. Numerous mRNA-based clinical applications are currently underway (as shown in Tables 1, 2, and 3), with many biotechnology companies establishing robust mRNA production pipelines. However, contaminants generated during the IVT process, particularly dsRNA, remain a significant hurdle. These contaminants can activate PKR signaling, suppress mRNA translation, trigger unwanted innate immune responses, and potentially lead to adverse side effects. Considerable efforts have been dedicated to improving mRNA purification technologies. RP-HPLC is one of the well-established methods for mRNA purification and dsRNA reduction. However, this technique requires costly equipment and is relatively time-consuming for large-scale mRNA purification and separation. In contrast, cellulose-based purification materials are more affordable and have lower operational costs but offer limited selectivity and recovery yield. As a result, they often need to be combined with other purification methods to achieve high-purity mRNA. As the mRNA therapeutics industry continues to grow, more biotechnology companies and research institutions are expected to enter the field, potentially driving further innovations in cost-effective IVT mRNA purification technologies and helping to overcome key barriers to large-scale manufacturing and clinical translation.

In addition to technical hurdles, the complex regulatory requirements also pose a considerable challenge for the mRNA industry. As we highlighted, the translation of mRNA therapeutics demands purification processes that can efficiently remove residual DNA templates, RNA polymerase, unincorporated nucleotides, truncated transcripts, and dsRNA by-products [146]. Standard downstream purification approaches such as tangential flow filtration and anion-exchange chromatography have been widely adopted for biomolecules purification; however, these methods are ineffective in dsRNA removal. Emerging techniques, such as affinity ligands specific to mRNA species or RNase III based dsRNA depletion offer increased selectivity but introduce novel materials that may face greater regulatory scrutiny and necessitate comprehensive safety assessments [147]. Innovations in IVT, including new or engineered RNA polymerases and transcription-enhancing additives, similarly must undergo thorough characterization and validation to demonstrate the sequence fidelity and complete removal of process byproducts and the added additives [148]. Regulatory authorities expect early engagement to align on purification methods and analytical assay requirements for impurity detection. While minor modifications to established purification or IVT protocols may proceed within several months, novel or complex purification platforms may require longer time for regulatory approval. Thus, strategic integration of scalable purification frameworks, proactive regulatory interactions, and investment in advanced analytical platforms are important to enabling the consistent manufacture of high-quality mRNA therapeutics for clinical and industrial applications [149].

Besides the mRNA purity, the therapeutic efficacy of mRNA also is critically affected by the chemical modifications of the mRNA nucleosides [150]. A pivotal modification is the incorporation of modified nucleosides such as N1-methylpseudouridine (m1Ψ) into the synthesized mRNA; this change is critical for RNA therapy as it substantially reduces the unintended/non-specific innate immunogenicity of mRNA molecules and mitigates adverse inflammatory responses [151]. This reduction in immunogenicity is achieved by dampening the activation of cellular sensors like Toll-like receptors. Furthermore, m1Ψ modification enhances the mRNA's stability and functional half-life and boosts its translational efficiency, leading to more robust and sustained production of the encoded therapeutic protein [150, 152]. Beyond m1Ψ, the field actively explores a diverse array of other chemical alterations, including various pseudouridine derivatives and 5-methylcytidine (m^5^C) [153–155]. The strategic selection and combination of these modifications are crucial for fine-tuning the mRNA's characteristics, such as its intracellular half-life, the kinetics of protein expression, and its overall safety profile, to tailor the mRNA molecule precisely for diverse RNA therapeutic applications [153].

The success of mRNA therapy also critically depends on the efficient and targeted delivery of therapeutic mRNA to specific cells, tissues, or organs, such as dendritic cells or the spleen for vaccine applications [156–158]. Naked mRNA is highly unstable in biological environments due to rapid degradation by nucleases, and its polyanionic nature further limits passive membrane penetration [159, 160]. In addition, intracellular delivery is hindered by endosomal entrapment and inefficient cytosolic release, which together substantially reduce translational efficiency [161, 162]. Lipid nanoparticles (LNPs) are currently the most clinically validated and widely used platform, demonstrating efficacy and real-world utility in COVID-19 vaccines and multiple therapeutic mRNA applications. Mechanistically, LNPs encapsulate mRNA within particles composed of ionizable lipids, phospholipids, cholesterol, and PEG-lipids. In acidic endosomes, the ionizable lipids become protonated, promoting endosomal membrane destabilization and facilitating endosomal escape, while the phospholipid, cholesterol, and PEG-lipid components contribute to particle stability, formulation properties, biodistribution, and cellular delivery [162]. These features collectively enable efficient cytosolic delivery and robust protein translation. Beyond LNPs, emerging platforms including polymeric nanoparticles [156, 163–166] and exosome-derived vesicles [167–169] are being developed to improve tissue specificity, reduce toxicity, and enable targeted delivery. Despite these advances, further optimization is still required to ensure scalable manufacturing, reproducible quality control, and consistent in vivo performance, which are essential for the full clinical translation of mRNA therapeutics.

## Conclusion

Together, mRNA technology offers unmatched adaptability compared to traditional platforms, particularly in the design of vaccine antigens and therapeutic molecules. This flexibility enables the inclusion of multiple mutant variants or even cross-species sequences within a single formulation, which is a significant advantage previously unattainable. The growing body of clinical data demonstrating safety and efficacy reinforces the transformative potential of mRNA therapeutics in modern medicine. In cancer immunotherapy, mRNA vaccines targeting the common tumor associated antigens and/or personalized neoantigens have shown therapeutic promise across numerous clinical trials. Researchers are increasingly combining conventional oncology treatments with novel strategies such as mRNA-based therapeutics, driving significant progress in cancer care. Beyond vaccines, mRNA therapies are poised to revolutionize not only immunotherapy but also protein replacement therapies. As research advances, the clinical adoption of more refined and effective RNA-based treatments is anticipated, enabling highly personalized and efficient therapeutic solutions.

## Data Availability

No datasets were generated or analysed during the current study.

## Abbreviations

ADARs: Adenosine deaminases acting on RNA enzymes, convert adenosine to inosine in double-stranded (ds) RNA
AEX: Anion exchange chromatography
AF4: Asymmetric flow field-flow fractionation
CAR: Chimeric antigen receptor
CARD: Caspase activation and recruitment domain
dsRNA: Double-stranded RNA
eIF2α: Eukaryotic initiation factor 2 alpha
ELISA: Enzyme-linked immunosorbent assay
eGFP: Enhanced green fluorescent protein
FDA: Food and Drug Administration
FLU: Influenza
GSDMD: Pore-forming protein gasdermin D
HPLC: High-performance liquid chromatography
HSV: Herpes simplex virus
IFN: Interferon
IRF3: Interferon regulatory factor 3
IVT: In vitro Transcription
LNPs: Lipid nanoparticles
LFSA: Lateral flow strip assay
LGP2: Laboratory of Genetics and Physiology 2 (a vital member of the RIG-I-like receptor (RLR) family of cytosolic RNA helicases)
MAVS: Mitochondrial antiviral signaling protein
MDA5: Melanoma differentiation-associated 5
mEPO: Murine erythropoietin
MPXV: Monkeypox virus
mRNA: Messenger RNA
NF-κB: Nuclear factor kappa-light-chain-enhancer of activated B cells
OASes: Oligoadenylate synthases
PKR: RNA-activated protein kinase
RIG-I: Retinoic acid-inducible gene I
RLRs: RIG-I-like receptors
RP-HPLC: Reverse phase high-performance liquid chromatography
RSV: Respiratory syncytial virus
sa-mRNA: Self-amplifying mRNA
ssRNA: Single-stranded RNA
SEC: Size-exclusion chromatography
TBK1: TRAF family member-associated NF-Kappa-B activator (TANK)-binding kinase 1
TFF: Tangential flow filtration
TLR3: Toll-like receptor 3
TRAF3: Tumor necrosis factor (TNF) receptor-associated factor 3
TRIF: Interleukin-1 receptor domain-containing adapter-inducing interferon-beta (a key cytosolic adapter protein critical for the innate immune response)
WMC: Wood-derived macroporous cellulose
